# Recruitment of LEF1 by Pontin chromatin modifier amplifies *TGFBR2* transcription and activates TGFβ/SMAD signalling during gliomagenesis

**DOI:** 10.1038/s41419-022-05265-y

**Published:** 2022-09-24

**Authors:** Xuexia Zhou, Xuebing Li, Run Wang, Dan Hua, Cuiyun Sun, Lin Yu, Cuijuan Shi, Wenjun Luo, Zhendong Jiang, Wenzhe An, Qian Wang, Shizhu Yu

**Affiliations:** 1 Department of Neuropathology, Tianjin Neurological Institute, Tianjin Medical University General Hospital, 300052 Tianjin, China; 2 Tianjin Key Laboratory of Injuries, Variations and Regeneration of the Nervous System, 300052 Tianjin, China; 3 Key Laboratory of Post-trauma Neuro-repair and Regeneration in Central Nervous System, Ministry of Education, 300052 Tianjin, China; 4 Tianjin Key Laboratory of Lung Cancer Metastasis and Tumor Microenvironment, Tianjin Lung Cancer Institute, Department of Lung Cancer Surgery, Tianjin Medical University General Hospital, 300052 Tianjin, China; 5 Department of Biochemistry and Molecular Biology, School of Basic Medical Sciences of Tianjin Medical University, 300070 Tianjin, China

**Keywords:** Cell invasion, CNS cancer

## Abstract

Synergies of transcription factors, chromatin modifiers and their target genes are vital for cell fate determination in human cancer. Although the importance of numerous epigenetic machinery for regulating gliomagenesis has been previously recognized, how chromatin modifiers collaborate with specific transcription factors remains largely elusive. Herein we report that Pontin chromatin remodelling factor acts as a coactivator for LEF1 to activate TGFβ/SMAD signalling, thereby contributing to gliomagenesis. Pontin is highly expressed in gliomas, and its overexpression paralleled the grade elevation and poor prognosis of patients. Functional studies verified its oncogenic roles in GBM cells by facilitating cell proliferation, survival and invasion both in vitro and in vivo. RNA sequencing results revealed that Pontin regulated multiple target genes involved in TGFβ/SMAD signalling. Intriguingly, we found that Pontin amplified TGFβR2 gene transcription by recruiting LEF1, thereby activating TGFβ/SMAD signalling and facilitating gliomagenesis. Furthermore, higher TGFβR2 expression conferred worse patient outcomes in glioma. To conclude, our study revealed that the Pontin-LEF1 module plays a crucial role in driving TGFβR2 gene transcription, which could be exploited to target TGFβ/SMAD signalling for anti-glioma therapy.

## Introduction

With the highest incidence among primary brain tumours, gliomas are highly lethal and have become a serious health threat worldwide [1]. The most frequent and malignant type of glioma, glioblastoma (GBM, WHO grade IV), is characterized by unlimited proliferation and persistent infiltration, which makes radical surgical resection almost impossible [2, 3]. Its easy relapse nature also causes a dismal prognosis for patients [2, 4]. Therefore, novel therapeutic strategies against malignant gliomas, especially GBM, are urgently needed.

Transforming growth factor-β (TGFβ) is an oncogenic factor in advanced cancers that regulates a vast majority of cellular processes, including proliferation, invasion, angiogenesis and immunosuppression [5–8]. Upon ligand binding, the TGFβ type II serine/threonine kinase receptor (TβRII) activates the type I receptor (TβRI), initiating an intracellular signalling cascade through phosphorylating specific receptor-regulated SMADs (R-SMADs) SMAD2 and SMAD3. Phosphorylation of SMAD2/3 facilitates their binding to SMAD4, and then the SMAD hetero-oligomer translocates into the nucleus, where it governs target gene transcription [6, 8, 9]. In the TGFβ/SMAD canonical signalling pathway, TβRII is the original receptor switch acting upstream of TβRI. Consequently, the content, stability and membrane distribution of TβRII are critical determinants of both the sensitivity and duration of the TGFβ response.

Pontin, officially named RUVBL1, is a chromatin modifier possessing both ATPase and DNA helicase activities [10–13]. It has been frequently found to be upregulated in multiple cancer types since its first discovery that its expression was upregulated in hepatocellular carcinoma [14, 15]. A key feature of Pontin is its participation in several conserved macromolecular protein complexes, which are involved in biological processes such as transcription regulation, chromatin remodelling, cell cycle/mitotic progression, DNA repair and cellular motility [16–20]. In transcriptional regulation, emerging evidence supports that Pontin functions as a coactivator for a variety of transcription factors, including androgen receptor (AR), T-cell factor (TCF) and hypoxia-inducible factor-1α (HIF-1α), in corresponding cellular contexts and signalling pathways [21–23]. Previously, we reported that Pontin exerted oncogenic roles in glioma by amplifying the E2F1 transcription response and promoting cell cycle progression [24]. However, its implication in gliomagenesis and the possible underlying mechanisms remain unclear.

In this study, we identified the Pontin chromatin remodelling factor as a key regulator of TβRII gene (TGFβR2) transcription, thus controlling TGFβ/SMAD signalling activity and TGFβ–dependent oncogenesis in GBM. Our findings illustrate the oncogenic roles of Pontin in glioma and suggest the possibility of Pontin as a promising prognostic marker and therapeutic target in glioma.

## Materials and methods

### Specimens from patients

Human clinical samples, including astrocytic gliomas (*n* = 120) and non-tumoral brain tissues (control, *n* = 20), were obtained from Tianjin Medical University General Hospital (TMUGH) with written consent. Following surgical resection, samples were immediately formalin-fixed and paraffin-embedded until continuous sections (5 μm thick) were prepared for IHC. Histopathological diagnoses of these specimens were completed by at least 2 neuropathologists according to the 2016 World Health Organization (WHO) criteria [3]. The genetic status of *IDH1/2* mutations was determined by Sanger sequencing. The clinicopathological characteristics of the 120 glioma patients enrolled in this study are provided in Supplementary Table 1.

### Immunohistochemical (IHC) staining

IHC staining was performed with mouse anti-human Pontin (catalogue SAB4200194; Sigma‒Aldrich, USA) and mouse anti-human Ki-67 primary antibodies (sc-23900, Santa Cruz Biotechnology, USA) following previously described procedures [25, 26]. Photographs were taken under a DM6000B microscope (Leica). Pontin or Ki-67 labelling indices (LIs) were determined by calculating the percentage of total cell numbers that were positively stained with Leica Image-Pro Plus 5.0 software.

### Cell culturing and reagents

The U87MG cell line was purchased from the American Type Culture Collection (ATCC, USA). The SNB19, U251, LN229, LN308 and U343 cell lines were obtained from the Chinese Academia Sinica Cell Repository. Isolated from a Chinese patient, the TJ905 cell line was maintained in our lab. The human non-tumoral control cell line UC2 was established by immortalizing astrocytes. All these cells were authenticated and tested for mycoplasma contamination. They were maintained in Dulbecco’s modified Eagle’s medium containing 10% foetal bovine serum (Gibco, USA) at 37 °C in a humidified incubator with 5% CO_2_. Recombinant human TGF-β1 was purchased from Invitrogen (USA), and LY2109761 (TGFβR2 inhibitor) was purchased from Selleck (China).

### Lentivirus and stable sub-cell line construction

Knockdown recombinant lentiviruses (puromycin resistance) expressing sh-Pontin (Pontin-kd) or negative control sh-NC (Control-kd) were obtained from GeneChem (China). The overexpression recombinant lentiviruses (blasticidin resistance) expressing Pontin (Pontin-ox), TGFβR2 or empty vector (Control-ox) were purchased from Applied Biological Materials (Canada). U87MG and U251 stable sub-cell lines were constructed by treatment with the corresponding lentivirus and selection under antibiotic (puromycin or blasticidin) resistance.

### Western blot and co-immunoprecipitation (Co-IP)

Western blot was performed according to our previously described protocols [26]. The primary antibodies were as follows: mouse anti-human Pontin (catalogue SAB4200194; Sigma‒Aldrich), mouse anti-human GAPDH (catalogue BM3876; Boster, China), goat anti-human TGFβRII (catalogue AF-241-NA; Biotechne, USA), rabbit anti-human CTGF (catalogue A11067; Abclonal, China), rabbit anti-human SMAD2/SMAD3 (catalogue 5678; Cell Signaling Technology, USA), rabbit anti-human phospho-SMAD2/SMAD3 (catalogue 8828; Cell Signaling Technology), and rabbit anti-human LEF1 (catalogue 2230; Cell Signaling Technology). Co-IP was conducted as previously described [24, 25]. Briefly, extracts from U87MG and U251 cells were immunoprecipitated with every 2 μg of mouse anti-flag tag antibody (catalogue F3165; Sigma‒Aldrich) for 4–6 h, followed by overnight incubation with protein A/G agarose beads (catalogue sc-2003; Santa Cruz Biotechnology). The conjunct complex was then washed, resuspended in protein sample buffer and subjected to SDS-PAGE. Then, target interacting proteins were revealed by immunoblot. Uncropped images for western blots are provided in the Supplementary Material.

### RNA extraction and quantitative RT-PCR (qRT-PCR)

Total RNA from clinical samples and various kinds of cells was isolated with TRIzol reagent (Invitrogen, USA) according to standard procedures. A total of 2.0 μg of RNA from each sample was subjected to reverse transcription (RT) by applying the M-MLV RT System (Promega, USA). The abundance of the target mRNA was measured by qRT-PCR employing GoTaq quantitative PCR Master Mix (Promega), and *ACTB*, which encodes β-actin, was used as the housekeeping gene. Primer sequences are provided in Supplementary Table 2. The 2^−ΔΔCt^ method was applied to calculate the relative mRNA expression (fold change).

### Cellular assays for proliferation, survival, migration and invasion

CCK-8 and 5-ethynyl-2’-deoxyuridine (EdU) staining assays were performed to evaluate cell proliferation as described previously [25, 27]. A colony formation assay was performed to measure the anchorage-dependent cell growth ability, which reflected cell survival. Standard procedures were followed as described previously [25]. Transwell assays were conducted to determine cell migration and invasion capacities as described previously [25, 26].

### Tumour xenograft assay

Four-week-old female BALB/C athymic nude mice (National Laboratory Animal Center, Beijing, China) were anaesthetized and transplanted intracranially with the indicated U87MG stable sub-cell lines (3 × 10^5^ cells per mouse, *n* = 5 for each group). Tumour growth was detected by bioluminescent imaging every week (Days 7, 14, 21 and 28). No randomization was used. No blinding was done. Tumour growth and survival situation of each mouse was recorded. Following sacrifice, brains were separated for haematoxylin and eosin (H&E) and IHC staining.

### Plasmids, transfection and luciferase reporter assay

The plasmid expressing full-length tagged human Pontin or LEF1 was constructed using the pcCMV-tag2b (Pontin) or pcDNA3.0-HA (LEF1) vector. TGFβ/SMAD signalling responsive CAGA12 reporter and *TGFBR2* promoter (−2000 to 0) reporter plasmids were constructed and synthesized by Genscript (Nanjing, China). Lipofectamine 3000 (Invitrogen, USA) was used for plasmid transfection. A luciferase reporter assay was performed according to the protocol provided by the manufacturer. Values are expressed as the means ± SDs from three independent experiments.

### Chromatin immunoprecipitation (ChIP) assay

Possible LEF1-binding sites in the *TGFBR2* promoter (−2000 to 0) were predicted by using JASPAR (http://jaspar.genereg.net/). An EZ-Magna-ChIP™ Kit (Millipore, USA) was used to conduct ChIP assays following the instructions provided by the manufacturer. Briefly, U87MG cells (5 × 10^6^) were first cross-linked with formaldehyde (1%), followed by chromatin digestion with micrococcal nuclease. The input reference of each experiment was saved as 2% aliquots of total lysates. Then, the other lysates were incubated with 8 μg of LEF1 antibody (catalogue ab137872; Abcam, USA) or control rabbit IgG (catalogue 12-370; Sigma‒Aldrich) at 4 °C overnight. NaCl plus proteinase K was used to reverse the cross-linked DNA. Finally, objective DNA was amplified from the recovered immunoprecipitated DNA at the previous step by qPCR. Sequences of the specific qPCR primers for the *TGFBR2* gene are shown in Supplementary Table 3.

### Immunofluorescence (IF) staining

Cells seeded on coverslips were fixed with paraformaldehyde (4%), permeabilized with Triton X-100 (0.2%), and stained as described previously [24]. Mouse anti-human Pontin antibody (catalogue SAB4200194; Sigma‒Aldrich) and rabbit anti-human LEF1 antibody (catalogue 2230; Cell Signaling Technology) were used as the primary antibodies. The secondary antibodies, including the TRITC-labelled goat anti-mouse IgG antibody (catalogue A16071) and FITC-labelled goat anti-rabbit IgG antibody (catalogue F-2765), were both purchased from Thermo Fisher Scientific. Cell nuclei were stained with DAPI. High-quality images were obtained under an FV-1000 laser-scanning confocal microscope (Olympus).

### RNA-seq experiment

Paired-end RNA sequencing of total RNA extracted from control (sh-NC) and Pontin knockdown (sh-Pontin) groups of U87MG cells was conducted using the Illumina HiSeq 2000 system. Sample preparation, library building, read mapping, transcriptome reconstruction, and data analysis for differentially expressed coding genes were performed sequentially, as we previously described [24, 25, 28].

### Statistical analysis

Statistical analyses were performed with SPSS 21.0 software (IBM, USA). Data are represented as the means ± SDs. Two-tailed Student’s *t-*test, one-way ANOVA, Pearson correlation analysis, Kaplan‒Meier analysis and log-rank test were applied to analyse the corresponding data. Statistical significance was assigned at *P* ≤ 0.05. All cellular experiments were performed at least three times with triplicate samples.

## Results

### Pontin is upregulated in gliomas, and its higher expression predicts worse patient outcome

**Fig. 1:**
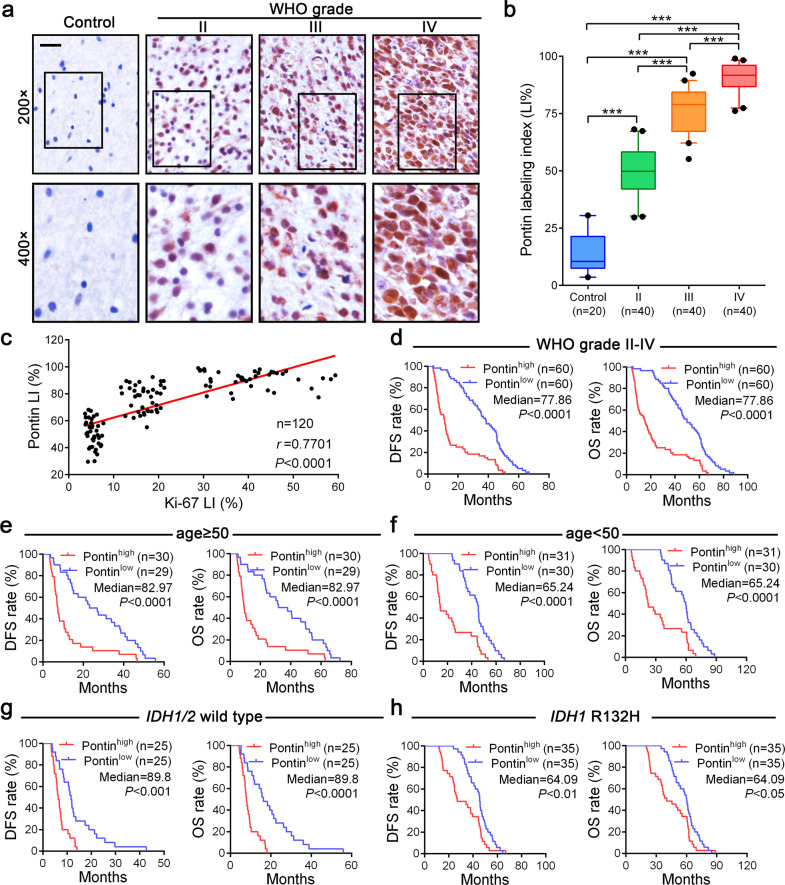
Pontin overexpression correlates with higher glioma grade and worse patient prognoses. **a** IHC of Pontin in control (non-tumoral brains, NBs) and graded glioma tissues. Scale bar, 20 μm. **b** Quantitative analysis of Pontin levels represented as IHC labelling indices (LIs [%]) among control (*n* = 20) and graded gliomas (*n* = 120, WHO grade II, III and IV). ***P < 0.001 by one-way ANOVA (Tukey’s post test). **c** Correlations of Pontin LIs with Ki-67 LIs in the 120 gliomas (Pearson correlation test). **d**–**h** Kaplan–Meier analyses of the DFS and OS. Patients were divided into high and low expression groups according to Pontin LI medians of the corresponding cohorts. *P*-values calculated by the log-rank (Mantel–Cox) test are shown.

Previously we found that Pontin mRNA was obviously upregulated in a large proportion of cancers, including GBMs and lower-grade gliomas (LGGs) [24]. To comprehensively evaluate its implication in gliomagenesis, a total of 20 non-tumoral brains and 120 gliomas with detailed patients’ clinical features were investigated in this study. IHC revealed that Pontin localized in the nucleus, and its higher expression paralleled the increase in glioma grade progression (Fig. 1a, b). In addition, a higher Pontin expression level was obviously associated with the proliferation index (Ki-67 LI; *r* = 0.7701, *P* < 0.0001; Fig. 1c), suggesting its importance in glioma cell proliferation. Kaplan‒Meier (KM) analyses revealed that higher Pontin expression predicted worse outcomes reflected by shorter disease-free survival (DFS; *P* < 0.0001) and overall survival (OS; *P* < 0.0001; Fig. 1d). When dividing these patients into cohorts according to age (age ≥ 50, age < 50), isocitrate dehydrogenase 1 and 2 (*IDH1/2*) genotype (*IDH1/2* wild-type, *IDH1* R132H) and Karnofsky Performance Status (KPS; <90, ≥90), we still observed an evident association between higher Pontin levels and worse patient prognosis (DFS: *P* < 0.01~0.0001; OS: *P* < 0.05~0.0001; Fig. 1e–h, Supplementary Fig. 1a, b). Moreover, Pontin upregulation was also significantly correlated with older age (*P* < 0.05), advanced grade (*P* < 0.0001), higher Ki-67 LI (*P* < 0.0001) and wild-type *IDH1/2* status (*P* < 0.0001; Table 1). The following Cox regression analysis indicated that the Pontin LI, similar to the Ki-67 LI, was also an independent predictor of DFS and OS in glioma patients (Supplementary Tables 4, 5). Western blot of Pontin expression among GBM cell lines verified its upregulation in GBM cell lines compared with that in the control UC2 immortal astrocyte cell line (Supplementary Fig. 1c). Taken together, these results clearly demonstrated that Pontin upregulation is highly involved in glioma progression, and Pontin might serve as a promising prognostic factor for predicting worse patient outcome.

**Table 1:** Relationship between Pontin expression and clinicopathological characteristics of the 120 glioma patients.

Feature	Non-increased (Pontin LI ≤ 77.86%) *n* = 60	Increased (Pontin LI > 77.86%) *n* = 60	Test of significance
Gender			
Male	35	36	χ^2^ = 0.034
Female	25	24	*P* = 0.85371
Age			
<50	37	24	χ^2^ = 5.635
≥50	23	36	*P* = 0.01761
Predominant side			
Left	26	31	
Right	31	25	
Middle	3	4	*P* = 0.54227
Predominant location			
Frontal lobe	40	35	
Temporal lobe	14	13	
Parietal lobe	4	5	
Occipital lobe	1	2	
Others	1	5	*P* = 0.48077
Grade			
II	34	6	
III	20	20	
IV	6	34	*P* < 0.0001
Ki-67 LI			
≤16.7	44	15	χ^2^ = 28.041
>16.7	16	45	*P* < 0.0001
IDH status			
Wild-type (IDH1/2)	12	38	χ^2^ = 23.177
Mutant type (IDH1 R132H)	48	22	*P* < 0.0001
KPS score			
<90	35	38	χ^2^ = 0.315
≥90	25	22	*P* = 0.57463

### Pontin promotes GBM cell proliferation and survival

**Fig. 2:**
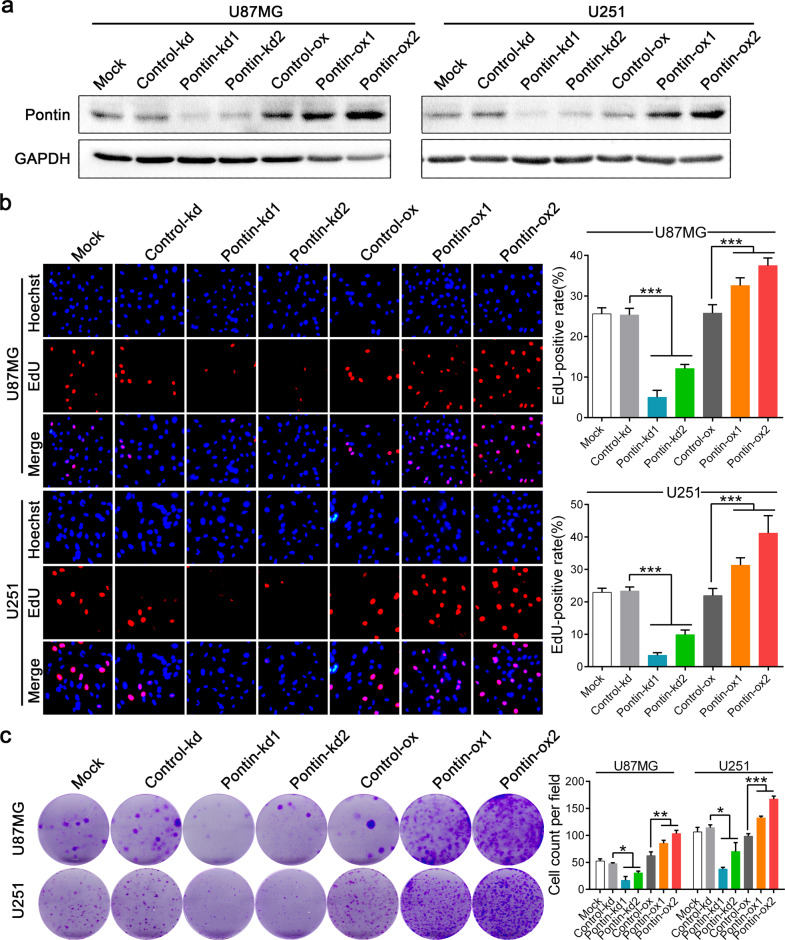
Pontin facilitates GBM cell proliferation and survival. **a** Western blot of Pontin in the extracts of the established Pontin-silenced or -overexpressed U87MG and U251 cells. Loading control, GAPDH. **b** Representative images of EdU staining (left) and the quantitative analysis of EdU-positive rates among the multiple groups (right). **c** Cell survival results reflected by colony formation assay. Data in **b, c** are expressed as means ± SDs, *n* = 3. ***P < 0.001 by one-way ANOVA (Dunnett’s post test).

To assess the effects of Pontin on gliomagenesis, we first performed cell proliferation assays (CCK-8 and EdU staining) and clonogenic survival assays (colony formation) in vitro. Two groups of Pontin knockdown sub-cell lines (kd1 and kd2) were constructed first by infecting U87MG and U251 cells with two independent shRNAs. Moreover, two groups of Pontin-overexpressing sub-cell lines (ox1, ox2) were obtained by infecting U87MG and U251 cells with 2 doses (MOI = 5, MOI = 10) of the Pontin overexpression lentivirus. Afterwards, we validated the efficient knockdown or overexpression of Pontin by qRT‒PCR and western blot (Supplementary Fig. 2a and Fig. 2a). Subsequent functional assays showed that, compared with the control or mock cells, Pontin knockdown inhibited GBM cell proliferation and survival, whereas Pontin overexpression facilitated GBM cell proliferation and survival (Supplementary Fig. 2b, Fig. 2b, c). These data confirm our previous conclusion that Pontin contributes to GBM cell growth in vitro.

### Pontin increases the invasive ability and tumorigenic potential of GBM cells

**Fig. 3:**
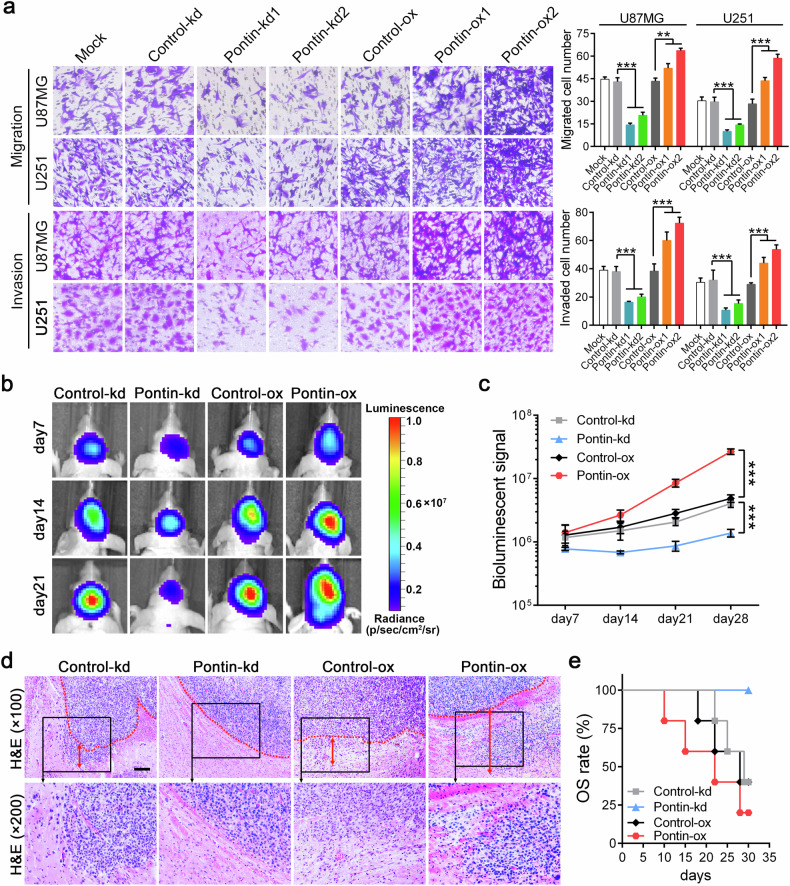
Pontin increases GBM cell migration, invasion and tumorigenic potential. **a** Representative images of transwell migration and invasion (left) and the comparison results (right). Data are expressed as means ± SDs, *n* = 3. **P < 0.01, ***P < 0.001 by one-way ANOVA (Dunnett’s post test). **b** Representative bioluminescent images revealing the xenografts in mice brain formed by indicated U87MG cells. **c** Quantification of the bioluminescence data monitored at Day 7, 14, 21 and 28 after transplantation. Data are expressed as means ± SDs, *n* = 5. ***P < 0.001 by one-way ANOVA (Dunnett’s post test). **d** Representative H&E staining images of the indicated xenografts. Red dotted lines labeled the junctions between xenografts and surrounding non-tumoral brain tissues. Invasion distances was indicated by red double-sided arrows. Scale bar, 50 μm (×100). **e** Survival curve of the tumour-bearing mice.

As is known, the infiltrating nature of GBM generally results in dismal prognoses [2–4]. We, therefore, sought to investigate whether Pontin affected GBM cell migration and invasion properties in vitro. The results from the transwell assays showed that Pontin knockdown suppressed, while its overexpression promoted, the migration and invasion of GBM cells (Fig. 3a). In the animal experiments, equal amounts of the abovementioned four groups of U87MG sub-cell lines (control-kd, Pontin-kd2, Control-ox and Pontin-ox2) were orthotopically transplanted into nude mice. Bioluminescence imaging showed that mice transplanted with Pontin-kd cells bore smaller tumours, whereas Pontin-ox cells bore larger tumours (Fig. 3b, c, Supplementary Fig. 3a). IHC verified that Pontin-kd xenografts retained Pontin silencing, while Pontin-ox xenografts expressed the highest level of Pontin (Supplementary Fig. 3a, b). The Ki-67 proliferation index exhibited an alteration tendency similar to that of Pontin (Supplementary Fig. 3a, b), indicating that Pontin affected GBM cell proliferation in vivo. Prominently, HE staining showed that xenografts formed by Pontin-kd cells barely exhibited any invasion abilities, while obvious invasion occurred in the Pontin-ox group (Fig. 3d). The KM results indicated that Pontin knockdown prolonged the OS of the mice, while its overexpression shortened the OS (Fig. 3e). Collectively, these results illustrated that Pontin possesses oncogenic roles in gliomagenesis by promoting cell proliferation and invasion.

### Pontin is associated with TGFβ signalling activation

**Fig. 4:**
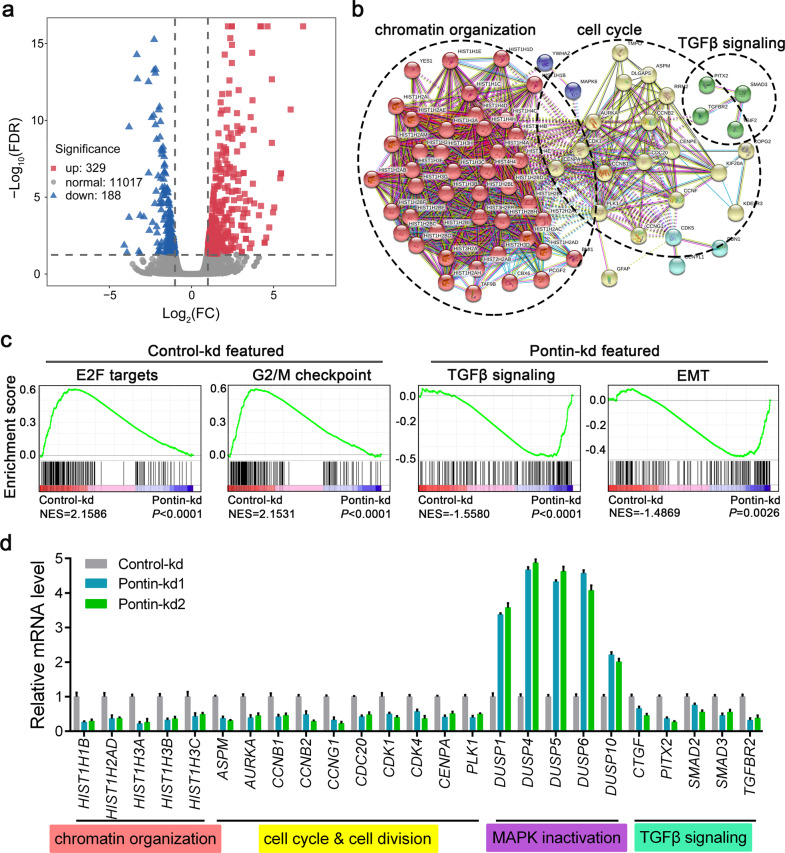
Global profiles of Pontin-regulated differential gene expression and cellular signalling in GBM cells. **a** Differential expressed coding genes affected by Pontin knockdown screened by RNA-seq (FC > 2, FDR < 0.05) were used to make the Volcano plot. **b** Downregulated genes incorporated in **a** were analyzed using STRING to plot the protein-protein association network with respective function annotation. **c** GSEA plots suggested a prominent correlation between Pontin and cellular signalling. **d** qRT-PCR confirmation of Pontin-regulated expression of genes involved in several biological processes or cellular signalling in indicated U87MG cells. Data are expressed as means ± SDs, *n* = 3.

Previously, we carried out RNA sequencing experiments using the control (Control-kd) and Pontin knockdown (Pontin-kd) U87MG sub-cell lines [24]. In retrospect of the differential expression of the coding genes between the two groups, we observed that those downregulated genes upon Pontin knockdown were mainly associated with functions such as chromatin organization, cell cycle and TGFβ signalling (Fig. 4a, b). In addition to the association of Pontin with E2F targets as described previously [24], gene set enrichment analysis (GSEA) also implied noteworthy correlations between Pontin and the G2/M checkpoint, TGFβ signalling, epithelial-mesenchymal transition (EMT), etc (Fig. 4c). The following qRT‒PCR validation confirmed the results from the RNA-seq analysis: Pontin promoted the expression of genes implicated in chromatin organization, cell cycle/cell division, and TGFβ signalling, whereas it suppressed the expression of genes associated with MAPK inactivation (Fig. 4d). It is well known that the TGFβ signalling pathway commonly exerts oncogenic potential and serves as an ideal therapeutic target for advanced cancers, including GBM [5, 6, 8, 29]. Therefore, through our preliminary screening by RNA-seq, we considered that Pontin might contribute to gliomagenesis by activating TGFβ signalling.

### Pontin is required to sustain TGFβ/SMAD signalling and is critical for TGFβ-induced GBM cell proliferation and invasion

**Fig. 5:**
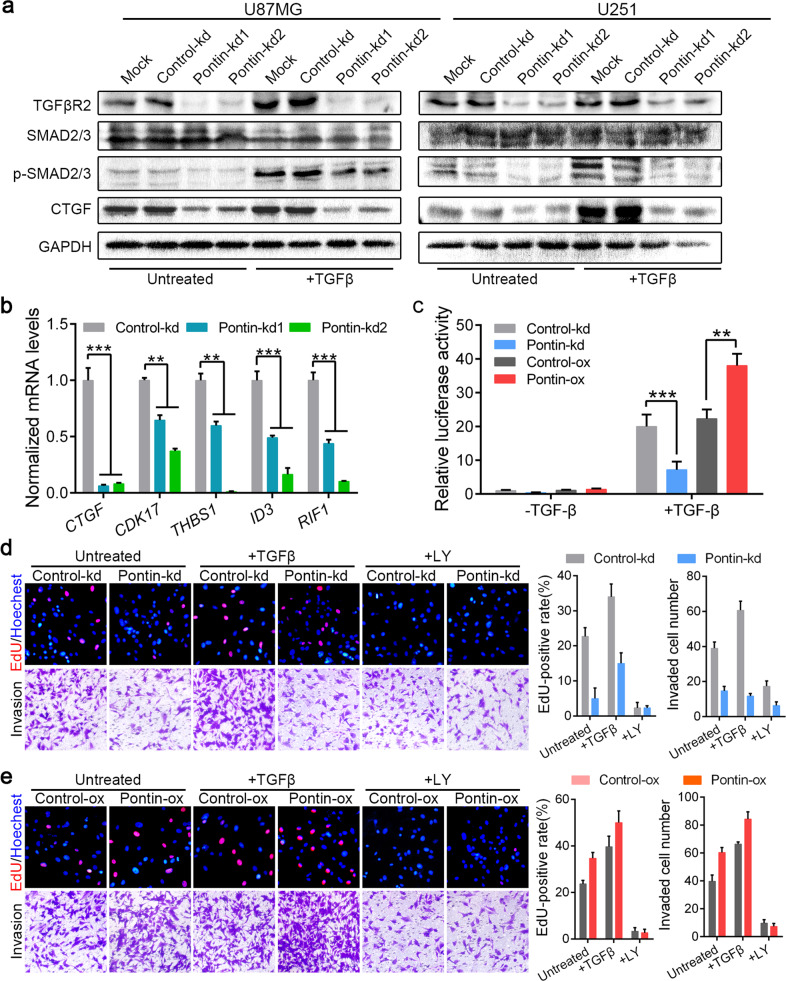
Pontin is required for TGFβ/SMAD signalling and critical for TGFβ-induced cell proliferation and invasion. **a** Western blot analyses of TGFβR2, p-SMAD2/3, SMAD2/3 and CTGF in the established Pontin-silenced U87MG and U251 cells without or with TGFβ (5 ng/ml) treatment overnight. Loading control: GAPDH. **b** qRT-PCR detection of TGFβ/SMAD target gene expression in the established Pontin-silenced U87MG cells. **c** Effect of Pontin knockdown or overexpression on the CAGA12-Luc transcriptional response induced by TGFβ (5 ng/ml) in U87MG cells. **d** Effect of Pontin knockdown on TGFβ- or LY-induced cell proliferation and invasion assessed by EdU staining and transwell invasion assays, respectively. Representative images (left) and the comparison results (right) were shown. **e** Effect of Pontin overexpression on TGFβ- or LY-induced cell proliferation and invasion assessed by EdU staining and transwell invasion assays, respectively. Representative images (left) and the comparison results (right) were shown. Data in **b**–**e** are expressed as means ± SDs, *n* = 3. **P < 0.01, ***P < 0.001 by one-way ANOVA (Dunnett’s post test).

The results above suggested that Pontin might affect TGFβ/SMAD signalling at the level of TGFβR2, since both RNA-seq analysis and qRT‒PCR validation confirmed that Pontin knockdown caused TGFβR2 mRNA reduction (Fig. 4b, d). As the first signalling molecule engaged by the TGFβ ligand, cell surface TGFβR2 is thought to be required for all TGFβ signalling responses. Western blot analysis revealed that Pontin knockdown led to downregulation of TGFβR2, phosphorylated SMAD2/3 (p-SMAD2/3) and CTGF, a well-known target of the TGFβ/SMAD signalling pathway in U87MG and U251 cells (Fig. 5a). TGFβ treatment activated the downstream SMAD-dependent signalling in control cells, manifesting as elevated p-SMAD2/3 and CTGF levels, whereas Pontin-silenced cells did not respond to or responded only slightly to TGFβ treatment, manifesting as lower p-SMAD2/3 and CTGF levels than the control groups (Fig. 5a and Supplementary Fig. 4). In contrast, Pontin-overexpressing cells exhibited higher levels of p-SMAD2/3 and CTGF, and they exhibited more activated TGFβ/SMAD signalling in response to TGFβ treatment, although the TGFβR2 inhibitor LY2109761 abrogated this effect (Supplementary Fig. 4). Evaluation of the expression of well-characterized direct transcriptional gene targets of TGFβ/SMAD signalling, i.e. *CTGF*, *CDK17*, *THBS1*, *ID3* and *RIF1*, by qRT‒PCR led to the same conclusion: Pontin knockdown led to TGFβ/SMAD signalling suppression (Fig. 5b). Consistently, Pontin knockdown decreased the luciferase activity of the TGFβ-induced SMAD3/4-driven CAGA12-luc transcriptional reporter [30]. In contrast, overexpression of Pontin increased the CAGA12-luc reporter activity (Fig. 5c).

Acting via specific receptors (TGFβR1 and TGFβR2), TGFβ stimulates GBM cell growth and invasion by activating a range of pathways and commonly leads to SMAD2/3 phosphorylation, which ultimately cooperates with SMAD4 mediator [29, 31]. Accordingly, Pontin-silenced U87MG cells exhibited reduced proliferation and invasion capacities (Fig. 5d). Upon knockdown of Pontin, the TGFβ-induced proliferation and invasion promotion were attenuated, while LY2109761 abolished the differences in proliferation and invasion between the control and Pontin knockdown groups (Fig. 5d). Ectopic expression of Pontin had the reverse effect: Pontin promoted GBM cell proliferation and invasion in response to TGFβ (Fig. 5e). Therefore, our findings suggested that Pontin is required to sustain TGFβ/SMAD signalling and is critical for TGFβ-induced GBM cell proliferation and invasion.

### Pontin acts as a coactivator for LEF1-dependent TGFβR2 gene transcription

**Fig. 6:**
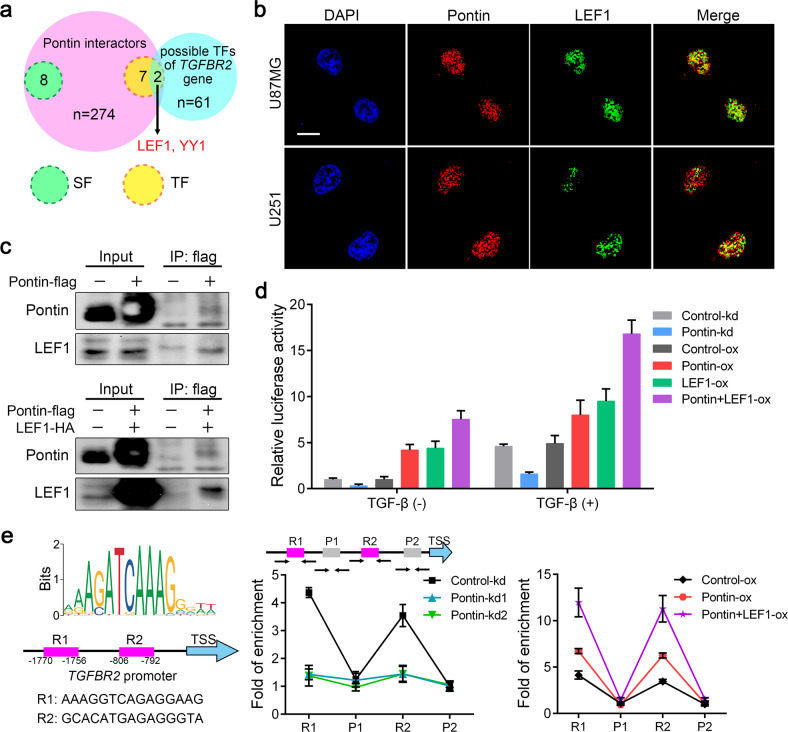
Pontin interacts with LEF1 to activate *TGFBR2* gene transcription in GBM cells. **a** Venn plot showed the overlapping of Pontin interacting proteins with possible transcription factors regulating *TGFBR2* gene expression. SF splicing factors, TF transcription factors. **b** Representative images showing that Pontin (red) colocalized with LEF1 (green) in U87MG and U251 cell nuclei. Scale bar, 10 μm. **c** Co-IP confirmation of the interaction between Pontin (flag-tagged) and endogenous LEF1 (upper) or HA-tagged LEF1 (bottom) in U87MG cells. **d** Effect of Pontin or LEF1 on the luciferase transcriptional response of the reporter driven by *TGFBR2* promoter (−2000 to 0). **e** LEF1-binding sites predicted in the *TGFBR2* promoter (left) and ChIP-qPCR analyses of the capacities for them to bind with LEF1 (right). Data in **d, e** are expressed as means ± SDs, *n* = 3.

Despite recent progress in elucidating the roles of TGFβ/SMAD signalling in cancers, the regulation of TGFβ type II receptor (TGFβR2) remains uncertain. From the above results, we observed that Pontin facilitated TGFβR2 expression at both the mRNA and protein levels (Figs. 4d and 5a). As Pontin could act as a coactivator for various transcription factors to control the transcription of target genes [15, 16, 21–23], we hypothesized that Pontin might regulate *TGFβR2* gene transcription by recruiting one possible transcription factor. Conjoint predictions of possible Pontin interactors by BioGRID (https://thebiogrid.org/; *n* = 274) and transcription factors (TFs) that might govern TGFβR2 gene transcription by JASPAR (http://jaspar.genereg.net/; *n* = 61) implied that LEF1 might interact with Pontin to control TGFβR2 gene transcription (Fig. 6a). IF detection revealed that Pontin colocalized with LEF1 in the nucleus (Fig. 6b). Co-IP results also indicated that Pontin indeed interacted with LEF1 in GBM cells (Fig. 6c).

We then performed luciferase reporter assays with the reporter derived from the wild-type *TGFβR2* promoter (−2000 to 0 from the transcription initiation site). Knockdown of Pontin attenuated the promoter activity. Consistent with this finding, overexpression of either Pontin or LEF1 increased the promoter activity, and co-overexpression of Pontin and LEF1 reinforced this increase by nearly twofold (Fig. 6d). TGFβ treatment obviously amplified the luciferase activities and still caused similar alterations among these groups (Fig. 6d).

Bioinformatics analysis of the *TGFβR2* promoter (−2000 to 0) by JASPAR revealed 2 potential LEF1-binding sites in the *TGFβR2* promoter (Fig. 6e, left; R1 and R2). ChIP‒qPCR verified that Pontin bound to both LEF1-binding sites in the TGFβR2 promoter. Upon Pontin knockdown, the enrichment at R1 and R2 was significantly decreased (Fig. 6e, middle). Specifically, Pontin overexpression increased the enrichment at R1 and R2; furthermore, co-overexpression of Pontin and LEF1 reinforced this increase (Fig. 6e, right). These results confirmed that Pontin can upregulate TGFβR2 gene transcription by recruiting LEF1 to the TGFβR2 promoter. In other words, Pontin acts as a coactivator for LEF1.

### Pontin induces GBM malignancy via TGFβR2, and higher TGFβR2 expression confers worse patient outcome

**Fig. 7:**
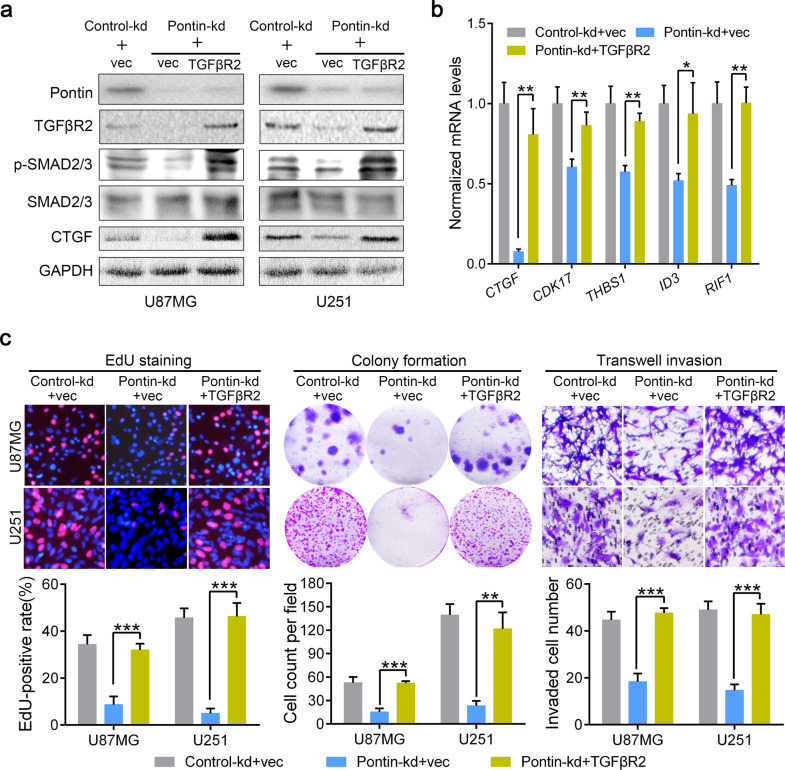
TGFβR2 efficiently mediates the GBM malignancy caused by Pontin. **a** Western blot analyses of Pontin, TGFβR2, p-SMAD2/3, SMAD2/3 and CTGF in the established TGFβR2-restored U87MG and U251 cells as indicated. **b** qRT-PCR detection of TGFβ/SMAD target gene expression in the established TGFβR2-restored U87MG cells. **c** The rescue effects of TGFβR2 on GBM cell proliferation (EdU staining, left), survival (colony formation, middle) and invasion (transwell invasion, right). Representative images from triple experiments (upper) and the quantification results (bottom) are shown. Data in **b, c** are expressed as means ± SDs, *n* = 3. *P < 0.05, **P < 0.01, ***P < 0.001 by one-way ANOVA (Dunnett’s post test).

To confirm that Pontin promotes gliomagenesis by enhancing *TGFβR2* gene transcription, we rescued TGFβR2 expression in the Pontin-silenced cells by stably transfecting empty vector (vec) or TGFβR2 plasmid into the control (Control-kd) or Pontin-silenced (Pontin-kd) cells (Fig. 7a). We found that TGFβR2 restoration also recovered TGFβ/SMAD signalling, as reflected by p-SMAD2/3, CTGF and TGFβ target gene expression (Fig. 7a, b). Cellular assays verified that restoration of TGFβR2 obviously rescued the inhibitory effects of Pontin silencing on GBM cell proliferation, survival and invasion (Fig. 7c), illustrating the significance of Pontin-regulated *TGFβR2* transcription in glioma progression.

**Fig. 8:**
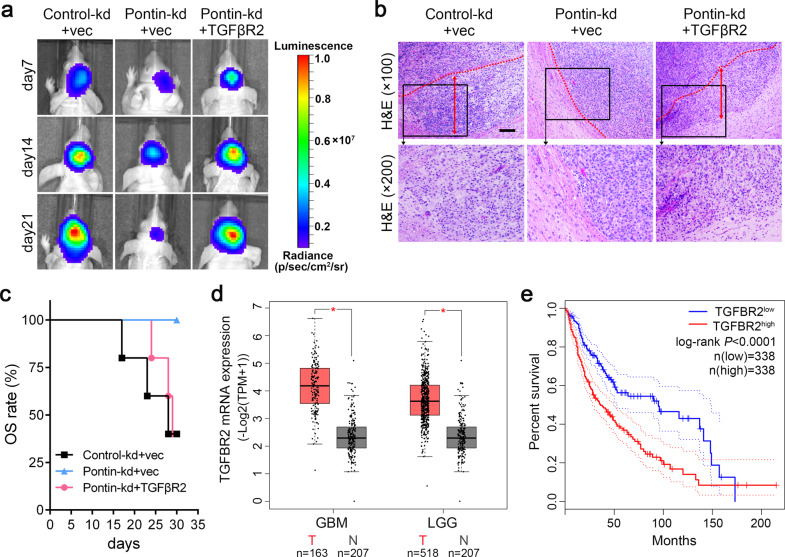
TGFβR2 restoration recovers xenograft growth and its higher levels indicate the worse outcomes in glioma patients. **a** Representative bioluminescent images reveal the xenografts in mice brain formed by indicated U87MG cells. **b** Representative H&E staining images of the indicated xenografts. Red dotted lines outline the junctions between xenografts and surrounding non-tumoral brain tissues. Invasion distances was indicated by red double-sided arrows. Scale bar, 50 μm (×100). **c** Survival curve of the glioma-bearing nude mice. **d** Box plots comparing *TGFBR2* mRNA expression between normal brains (N) and tumours (T) in GBM and LGG (lower-grade glioma) using the data from GEPIA dataset. **e** Kaplan–Meier analyses the OS of all the glioma patients (GBM and LGG) from the GEPIA dataset. Patients were divided into high and low expression groups according to *TGFBR2* expression medians of the corresponding cohorts. *P*-values calculated by the log-rank (Mantel–Cox) test are shown.

Subsequently, we verified whether TGFβR2 mediated the tumour-promoting effects of Pontin in vivo. The aforementioned three groups of U87MG sub-cell lines (Control-kd+vec, Pontin-kd+vec and Pontin-kd+TGFβR2) were transplanted into nude mice. In accordance with the above cellular results, TGFβR2 abrogated the suppressive effects of Pontin silencing on xenograft growth and invasion and prolonged the survival of the mice as the controls (Fig. 8a–c).

We then examined the expression of TGFβR2 in human gliomas by analysing the online GEPIA data. The results in Fig. 8d indicated that *TGFβR2* mRNA was increased in both GBMs and LGGs in comparison to corresponding normal brains. KM analysis showed that patients possessing *TGFβR2* overexpression had remarkably shorter OS (Fig. 8e). Collectively, these data concluded that Pontin increases GBM malignancy by upregulating TGFβR2, whose overexpression confers worse patient prognosis for glioma patients.

## Discussion

**Fig. 9:**
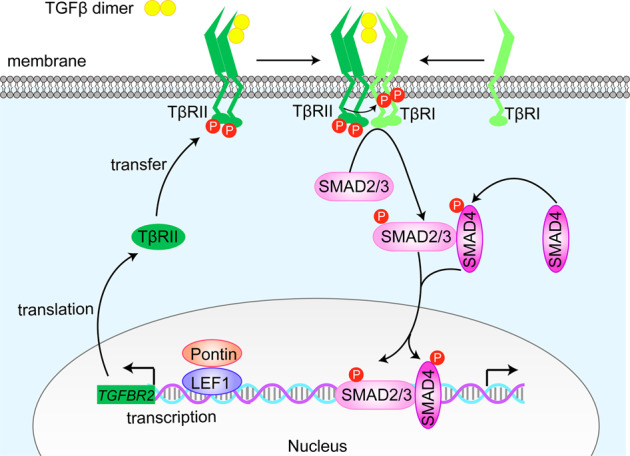
Schematic illustration of the mechanism by which Pontin promotes gliomagenesis through amplifing TGFBR2 transcription and activating TGFβ/SMAD signalling.

The present work is an extension of the Pontin chromatin modifier in GBM study and builds upon the first preliminary investigation by Wang et al. [24]. In this study, we confirmed the upregulation of Pontin in glioma and further verified its correlation with glioma progression and patient prognosis. More convincing evidence proved that Pontin promoted gliomagenesis by facilitating cell proliferation and invasion. Mechanistically, Pontin amplified *TGFβR2* gene transcription by recruiting LEF1, thereby activating TGFβ/SMAD signalling and contributing to GBM malignancy (Fig. 9).

Accumulating evidence supports Pontin upregulation in malignancies [15], but few studies have evaluated the clinical significance of Pontin in cancers. Using the public online resources, we previously found the overexpression of Pontin and its poor prognosis predicting significance for glioma patients [24]. Herein, by conducting IHC of 20 non-tumoral brains and 120 gliomas with detailed clinical features, we confirmed the upregulation of Pontin in glioma and verified its positive correlation with advanced glioma grade, high proliferation index and short patient survival. Therefore, increased Pontin expression might serve as a promising prognostic biomarker in gliomas.

Implicated in a wide range of biological processes, Pontin is known to be crucial for oncogenesis by participating in transcription control, chromatin remodelling, cell cycle/mitotic progression, DNA repair and cellular motility [16–20]. In our previous study, we showed that Pontin facilitated glioma progression by accelerating cell cycle progression and cell growth. Nevertheless, in vivo experiments were lacking [24]. Herein, we provide solid in vitro and in vivo evidence that Pontin exerts oncogenic roles in GBM by enhancing cell proliferation, survival and invasion.

Pontin is well recognized for its involvement in transcription regulation in numerous cellular contexts [16, 21–23]. As we have shown previously, Pontin interacted with E2F1, increased E2F1-dependent transcription and promoted the expression of E2F1 targets in GBM cells [24]. We believe that the tight interaction of Pontin-E2F1 to activate downstream target transcription represents a vital pathway but not the only one account for the oncogenic roles of Pontin. Data mining of the RNA-seq results derived from Pontin-silenced cells and control cells raised another possibility, which is that Pontin might positively affect TGFβ/SMAD signalling. Functional studies revealed that Pontin was required to sustain TGFβ/SMAD signalling and was critical for TGFβ-induced GBM cell proliferation and invasion.

TGFβ signalling is hyperactivated in advanced cancers [7, 8, 31]. TGFβR2 is the first signalling molecule engaged by the TGFβ ligand, and its cell surface content determines the activating degree of all TGFβ signalling responses. Our careful exploration found that Pontin facilitated TGFβR2 mRNA and protein expression concurrently. More detailed experiments revealed that Pontin could induce *TGFβR2* gene transcription by recruiting LEF1 to the TGFβR2 promoter. In brief, Pontin might be a coactivator for LEF1-dependent *TGFβR2* gene transcription. Importantly, subsequent rescue experiments clearly proved that TGFβR2 was a key factor mediating the oncogenic roles of Pontin in GBM cells since its restoration effectively restored TGFβ/SMAD signalling, as well as GBM malignancy both in vitro and in vivo.

Lymphoid enhancer-binding factor-1 (LEF1) belongs to the TCF/LEF1 family of high mobility transcription factors. It principally participates in the Wnt/β-catenin signalling pathway [32]. A couple of studies have reported LEF1 overexpression in glioma, lung cancer and oral squamous cell carcinoma. Abnormal LEF1 overexpression was strongly linked to tumorigenesis and might predict poor patient prognosis [33–35]. Specifically, LEF1 promotes cancer cell proliferation, viability and EMT, a key characteristic of cancer cell migration and invasion [32, 36]. Our results clearly showed that Pontin cooperated with LEF1 to promote *TGFβR2* gene transcription, thereby activating TGFβ/SMAD signalling and enhancing GBM malignancy. The linkage of LEF1 with TGFβ/SMAD signalling has also been observed in oesophagal squamous cell carcinoma by Zhao et al. [37].

## Conclusion

In summary, our comprehensive findings on Pontin elucidate its functions and molecular mechanism in promoting gliomagenesis. Collaboration with LEF1 to activate *TGFβR2* gene transcription is a vital mechanism underlying the tumour-promoting capacities of Pontin. Together, our results verify that Pontin acts as a crucial oncogene by recruiting LEF1 to amplify *TGFβR2* transcription and activate TGFβ/SMAD signalling. Our investigation reveals the probability of targeting Pontin for anti-glioma therapy.

### Supplementary information

Supplementary materials

Reproducibility checklist

language editing certificate

## Data Availability

All data are available upon request of the corresponding author.
