# Correction: Recruitment of LEF1 by Pontin chromatin modifier amplifies *TGFBR2* transcription and activates TGFβ/SMAD signalling during gliomagenesis

**DOI:** 10.1038/s41419-024-06824-1

**Published:** 2024-07-09

**Authors:** Xuexia Zhou, Xuebing Li, Run Wang, Dan Hua, Cuiyun Sun, Lin Yu, Cuijuan Shi, Wenjun Luo, Zhendong Jiang, Wenzhe An, Qian Wang, Shizhu Yu

**Affiliations:** 1 Department of Neuropathology, Tianjin Neurological Institute, Tianjin Medical University General Hospital, 300052 Tianjin, China; 2 Tianjin Key Laboratory of Injuries, Variations and Regeneration of the Nervous System, 300052 Tianjin, China; 3 Key Laboratory of Post-trauma Neuro-repair and Regeneration in Central Nervous System, Ministry of Education, 300052 Tianjin, China; 4 Tianjin Key Laboratory of Lung Cancer Metastasis and Tumor Microenvironment, Tianjin Lung Cancer Institute, Department of Lung Cancer Surgery, Tianjin Medical University General Hospital, 300052 Tianjin, China; 5 Department of Biochemistry and Molecular Biology, School of Basic Medical Sciences of Tianjin Medical University, 300070 Tianjin, China

**Keywords:** Cell invasion, CNS cancer

Correction to: *Cell Death & Disease*

10.1038/s41419-022-05265-y, published online 24 September 2022

There is an error in the usage of images in Fig. 3a, which might lead to misunderstandings for readers.

The revised figure with the corrected images are resubmitted. This correction will not affect any of the salient points of the work, the interpretation of the data and the conclusions.

Incorrect Fig. 3a

Correct Fig. 3
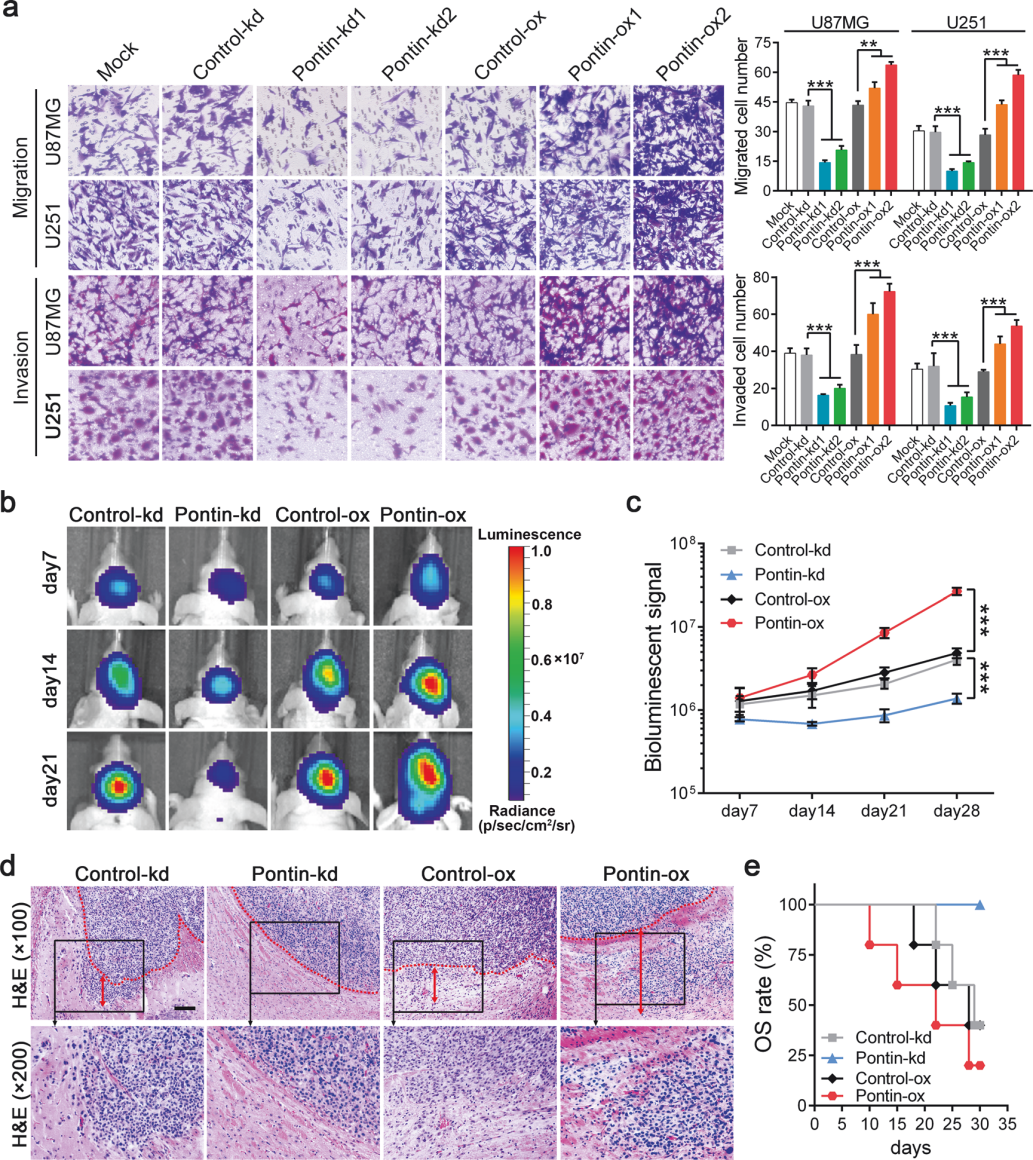

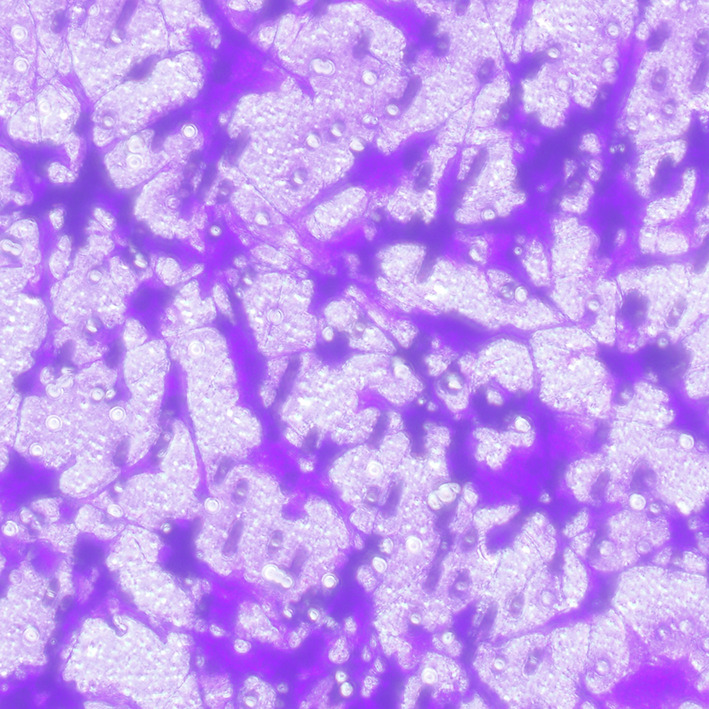

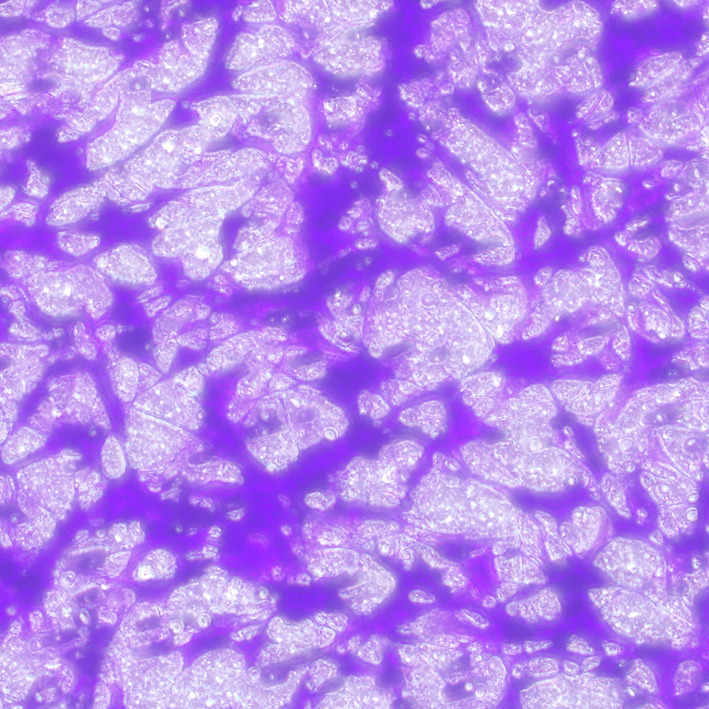


The original article has been corrected.

