# Supplementary material for: Recruitment of LEF1 by Pontin chromatin modifier amplifies TGFBR2 transcription and activates TGFβ/SMAD signalling during gliomagenesis
Source: Cell Death Dis. 2022 Sep 24;13(9):818. doi: 10.1038/s41419-022-05265-y (PMC9509381; doi:10.1038/s41419-022-05265-y)

Supplementary information

Supplementary Tab. 1~Tab. 5

Supplementary Fig. 1~Fig. 4

Supplementary Tab. 1 Clinicopathological characteristics of the 120 glioma patients enrolled in this study

Feature	WHO Grade		
	II (n=40)	III (n=40)	IV (n=40)
Gender			
Male	22	22	27
Female	18	18	13
Age (Year, Mean±SD)	42.73±11.88	47.68±15.24	55.68±13.05
Age < 50	29	22	10
Age ≥ 50	11	18	30
Predominant side			
Left	20	17	20
Right	17	21	18
Middle	3	2	2
Predominant location			
Frontal lobe	30	26	19
Temporal lobe	6	8	13
Parietal lobe	1	3	5
Occipital lobe	1	1	1
Others	2	2	2
IDH1/2 status			
Wild type (*IDH1/2*)	5	7	38
Mutant type (*IDH1* R132H)	35	33	2
KPS score			
< 90	23	24	26
≥ 90	17	16	14

Abbreviation: SD, Standard deviation; KPS, Karnofsky performance score.

Supplementary Tab. 2 Sequences of the qRT-PCR primers

Name	Forward sequence (5'-3')	Reverse sequence (5'-3')
Pontin	5'-CGGGTGTGCTGTTTGTTGAT-3'	5'-AAGATGACGATGGGAGCGATA-3'
ACTB	5'-GATCATTGCTCCTCCTGAGC-3'	5'-ACTCCTGCTTGCTGATCCAC-3'
HIST1H1B	5'- GCTTCTAAGGAGCGCAATGG-3'	5'-CTTGAGGCCCAGCTTAATGC-3'
HIST1H2AD	5'-CGCCCGCGACAACAAG-3'	5'-CGCCCTGAGCAATTGTGACT-3'
HIST1H3A	5'-TAAGGCAGCCCGCAAAAG-3'	5'-CAGGCGCTGGAAAGGTAGTT-3'
HIST1H3B	5'- CGCCTGGTGCGAGAAATC-3'	5'-TTAGCATGGATGGCGCAAA-3'
HIST1H3C	5'-AAATCGCCCAGGACTTCAAA-3'	5'-GAATAGCGCACAGATTGGTGTCT-3'
ASPM	5'-TAGGCGGTTAATTGTTCGAAAAG-3'	5'-ATTCGAAGCCACAAAGGATTGT-3'
AURKA	5'- GCCATCGGCACCTGAAAATA-3'	5'-CCCACTGCCTCTTTTTTGATTC-3'
CCNB1	5'-GCACTTCCTTCGGAGAGCAT-3'	5'-GGAGGAAAGTGCACCATGTCA-3'
CCNB2	5'-GCATGCGTGCCATCCTAGT-3'	5'-CTGGCTGAACCTGTAAAAATCGA-3'
CCNG1	5'- GGCCTCAGAATGACTGCAAGA-3'	5'-TGGGCTGTACCTTCATTTTAGACA-3'
CDC20	5'-CGCCTGAAATCCGAAATGAC-3'	5'-AGCTTGCACTCCACAGGTACAC-3'
CDK1	5'-CCAATAATGAAGTGTGGCCAGAA-3'	5'-ATGCTAGGCTTCCTGGTTTCC-3'
CENPA	5'-TCACTCGTGGTGTGGACTTCA-3'	5'-AACTCGGCCTGCATGTAAGG-3'
PLK1	5'-TTCGTGTTCGTGGTGTTGGA-3'	5'-GGCTCAGTCAGGGCTTTCC-3'
DUSP1	5'- ACGAGGCCTTTGAGTTTGTGA-3'	5'-CAGCACCTGGGACTCAAACTG-3'

DUSP4	5’-CTCGGACTGCCCAAACCA-3’	5’-CCTTCACGGCATCGATGTACT-3’
DUSP5	5’- CAAGGTCCTGGTCCACTGTGA-3’	5’-GCGGAACTGCTTGGTCTTCA-3’
DUSP6	5’-GCAGCGACTGGAACGAGAAT-3’	5’-AGGGAGAACTCGGCTTGGAA-3’
DUSP10	5’- TTCCTGTTCCTTGGCAATGAG-3’	5’-GGAAGATGAGTGGTGACGTTGA-3’
CTGF	5’-ACGAGCCCAAGGACCAAAC-3’	5’-GAACAGGCGCTCCACTCTGT-3’
PITX2	5’- CATGTCCACACGCGAAGAAA-3’	5’-CCCGACGATTCTTGAACCAA-3’
SMAD2	5’-CAGGGTTTTGAAGCCGTCTATC-3’	5’-ACCGTCTGCCTTCGGTATTCT-3’
SMAD3	5’- CGCAGAACGTCAACACCAAGT-3’	5’-ACAGGCGGCAGTAGATGACAT-3’
TGFBR2	5’-ACCACTGGGAGTTGCCATATCT-3’	5’-TCCCAGGTTGAACTCAGCTTCT-3’
CDK17	5’-AGCCAAGTCAGTTCCCACAAA-3’	5’-CTCCGAGGAACCAAGAAGCA-3’
THBS1	5’- TTTGCCGGCGTGAAGTGTA-3’	5’-AGGCACTTCTTTGCACTCATCA-3’
ID3	5’-GAGCTTGCTGGACGACATGA-3’	5’-CGATGACGCGCTGTAGGATT-3’
RIF1	5’- CTCCTTTGGCTTCTCCGTCTAC-3’	5’-CTGCTTGGTATATTGGATCTGCAA-3’

Supplementary Tab. 3 Sequences of the CHIP-qPCR primers

Name	Forward sequence (5’-3’)	Reverse sequence (5’-3’)
R1	5’-CAAGATCTACTCATGGAGGTG-3’	5’-TATGCCTCTGTAATCCTCCTACCC-3’
P1	5’-GGATCTGGTTTTTATCTTCTC-3’	5’-TTGTGTAGGAGTCTCCCACGAGT-3’
R2	5’-ACATGTACACCAGGAATGTCTTG-3’	5’-CGAAGTGATAGTGGTGGGAAATG-3’
P2	ATAGACACAACTGAAGCACAAA	5’-CCTAGTAGATCAGAACATCTGC-3’

Supplementary Tab. 4 Multivariate analysis for DFS and OS in patients with gliomas

Factors	DFS		OS	
	HR (95%CI)	*P*	HR (95%CI)	*P*
Gender	1.087 (0.722-1.636)	0.691	1.102 (0.733-1.657)	0.639
Age	0.995 (0.979-1.010)	0.488	0.994 (0.980-1.010)	0.469
Predominant side	0.849 (0.588-1.225)	0.381	0.776 (0.536-1.123)	0.178
Predominant location	1.122 (0.849-1.483)	0.418	1.017 (0.766-1.349)	0.909
IDH status	0.152 (0.065-0.351)	<0.0001	0.084 (0.038-0.188)	<0.0001
KPS	0.983 (0.956-1.011)	0.232	0.981 (0.953-1.009)	0.189
Pontin LI	0.940 (0.922-0.959)	<0.0001	0.935 (0.916-0.954)	<0.0001
Ki-67 LI	1.415 (1.329-1.508)	<0.0001	1.473 (1.377-1.575)	<0.0001

Supplemental Tab. 5 Univariate analysis for DFS and OS in patients with gliomas

Factors	DFS		OS	
	HR (95%CI)	*P*	HR (95%CI)	*P*
Gender	0.920 (0.637-1.329)	0.656	0.925 (0.640-1.336)	0.677
Age	1.022 (1.008-1.036)	0.002	1.021 (1.007-1.035)	0.003
Predominant side	0.963 (0.713-1.301)	0.806	0.953 (0.705-1.289)	0.756
Predominant location	1.291 (1.022-1.630)	0.032	1.283 (1.016-1.620)	0.037
Grade	5.557 (3.915-7.889)	<0.0001	5.622 (3.932-8.038)	<0.0001
IDH status	0.089 (0.055-0.146)	<0.0001	0.086 (0.052-0.141)	<0.0001
KPS	0.994 (0.972-1.016)	0.585	0.994 (0.972-1.016)	0.582
Pontin LI	1.039 (1.027-1.052)	<0.0001	1.038 (1.025-1.050)	<0.0001
Ki-67 LI	1.249 (1.206-1.293)	<0.0001	1.252 (1.209-1.297)	<0.0001

Supplementary Fig. 1

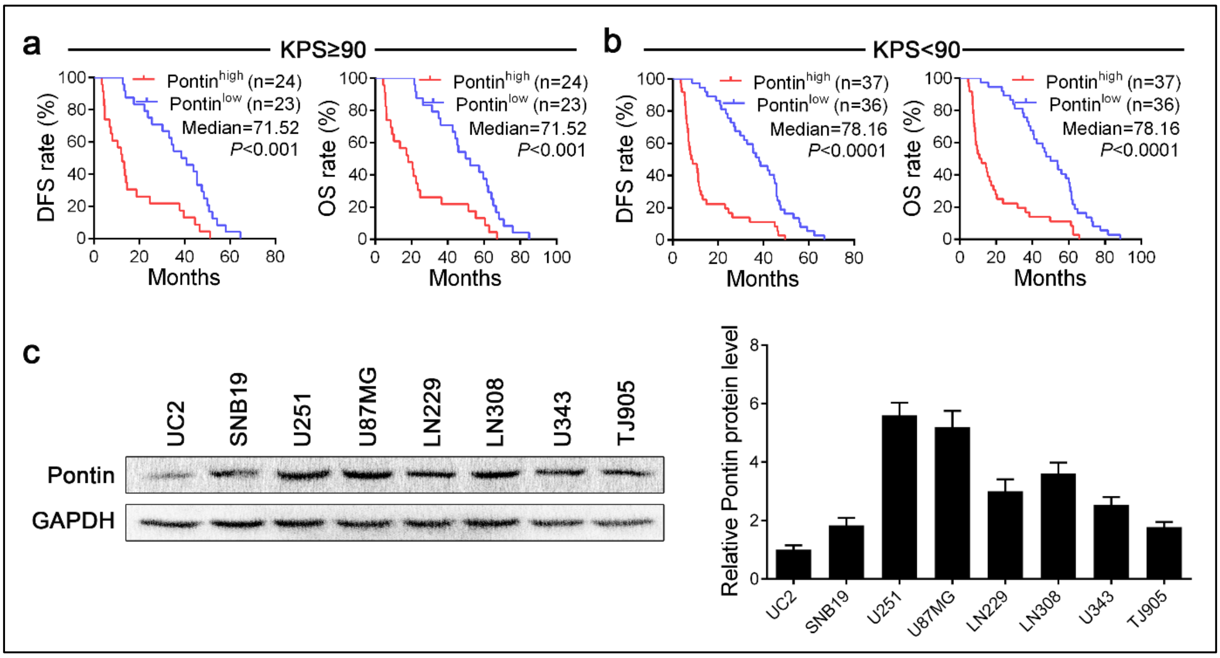

Supplementary Fig. 1 High Pontin levels correlate with poor prognoses of glioma patients. **a** Kaplan-Meier analyses of the DFS and OS of the glioma patients with similar KPS (≥90 or <90). Patients were stratified into high and low expression groups using the medians of the Pontin LIs of the corresponding cohorts. *P* values of the log-rank (Mantel-Cox) tests are presented. **b** Western blot of Pontin expression (left) and the quantification result (right) among 7 GBM cell lines and UC2 (an immortal astrocyte cell line). Loading control, GAPDH. Data are expressed as mean ± SD, n=3.

Supplementary Fig. 2

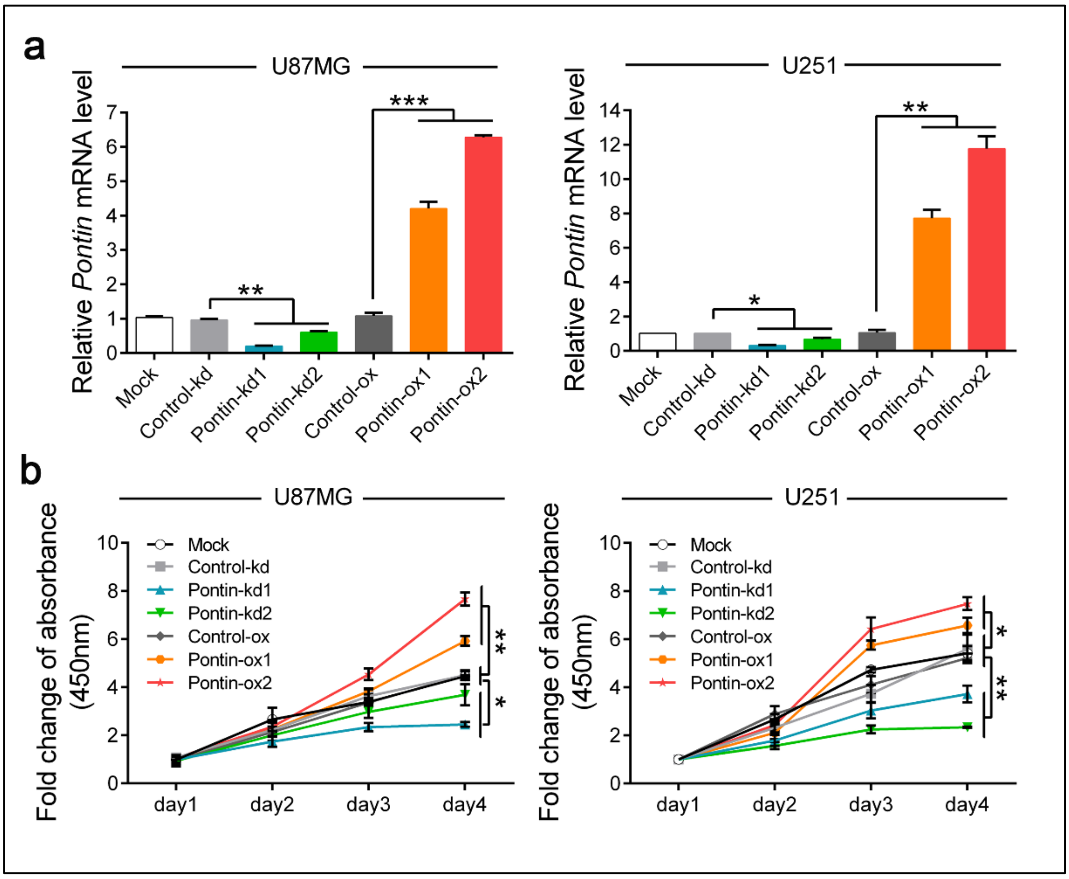

Supplementary Fig. 2 Pontin promotes GBM cell proliferation in vitro.

a qRT-PCR detection of Pontin expression in the extracts of the established Pontin-silenced or –overexpressed U87MG and U251 cells as indicated. **b** Growth curves of the established Pontin-silenced or –overexpressed U87MG and U251 cells as indicated.

Supplementary Fig. 3

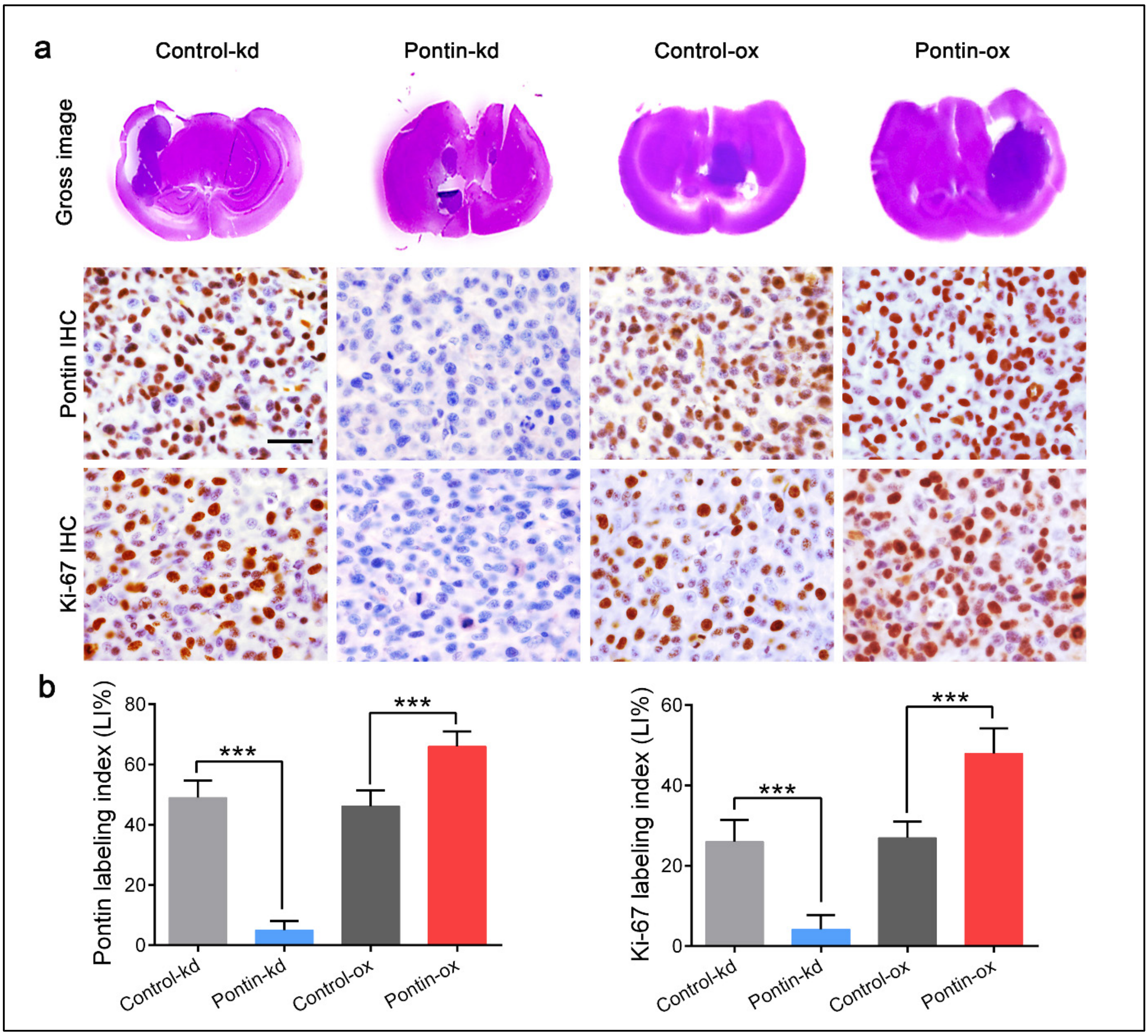

Supplementary Fig. 3 In vivo study of Pontin knockdown or overexpression on the intracranial xenograft formation using U87MG cells.

a H&E staining brain slices of the nude mice bearing glioma xenografts and IHC of Pontin, Ki-67 in outgrowing tumor slices. Images of representative tumors are shown. Scale bars for IHC, 20 μm. **b** Quantification results of the IHC staining in (A) for Pontin (left) or Ki-67 (right) expression levels among the four groups as indicated. ****P*<0.001 by 1-way ANOVA (Tukey's post test).

Supplementary Fig. 4

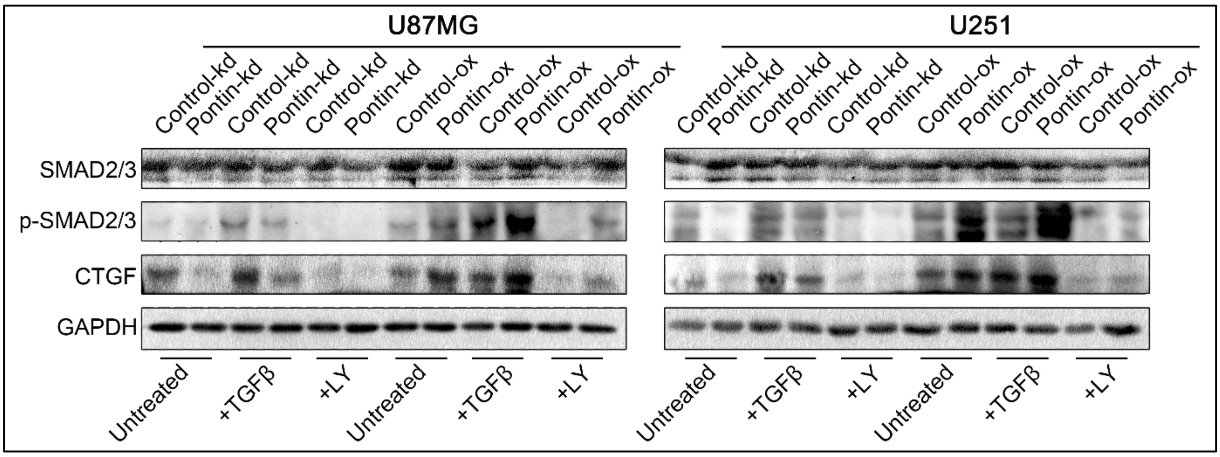

Supplementary Fig. 4 Pontin activates TGFβ/SMAD signaling in GBM cells.

Western blot analyses of p-SMAD2/3, SMAD2/3 and CTGF in the established Pontin-silenced or –overexpressed U87MG and U251 cells without or with TGFβ/LY treatment overnight.

Figure 2

a

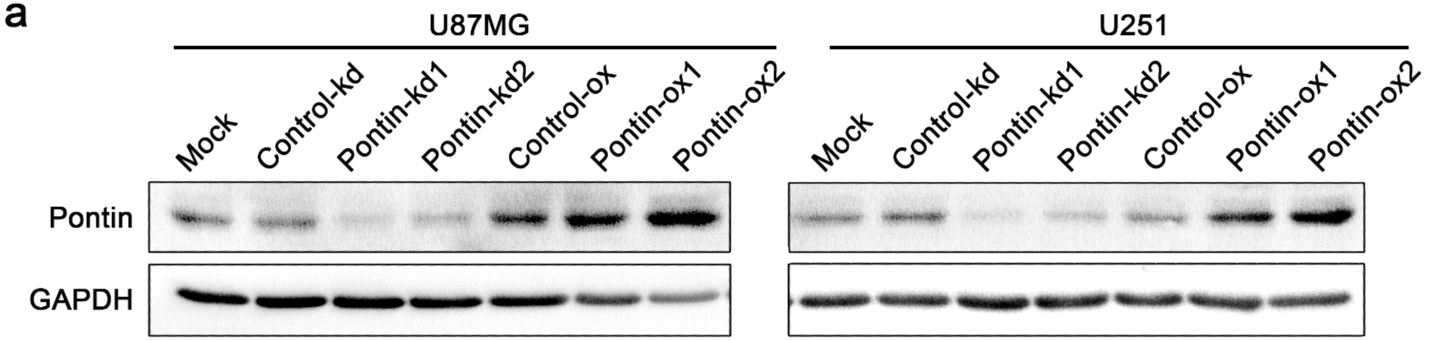
U87MG
U251
Mock
Control-kd
Pontin-kd1
Pontin-kd2
Control-ox
Pontin-ox1
Pontin-ox2
Mock
Control-kd
Pontin-kd1
Pontin-kd2
Control-ox
Pontin-ox1
Pontin-ox2
Pontin
GAPDH

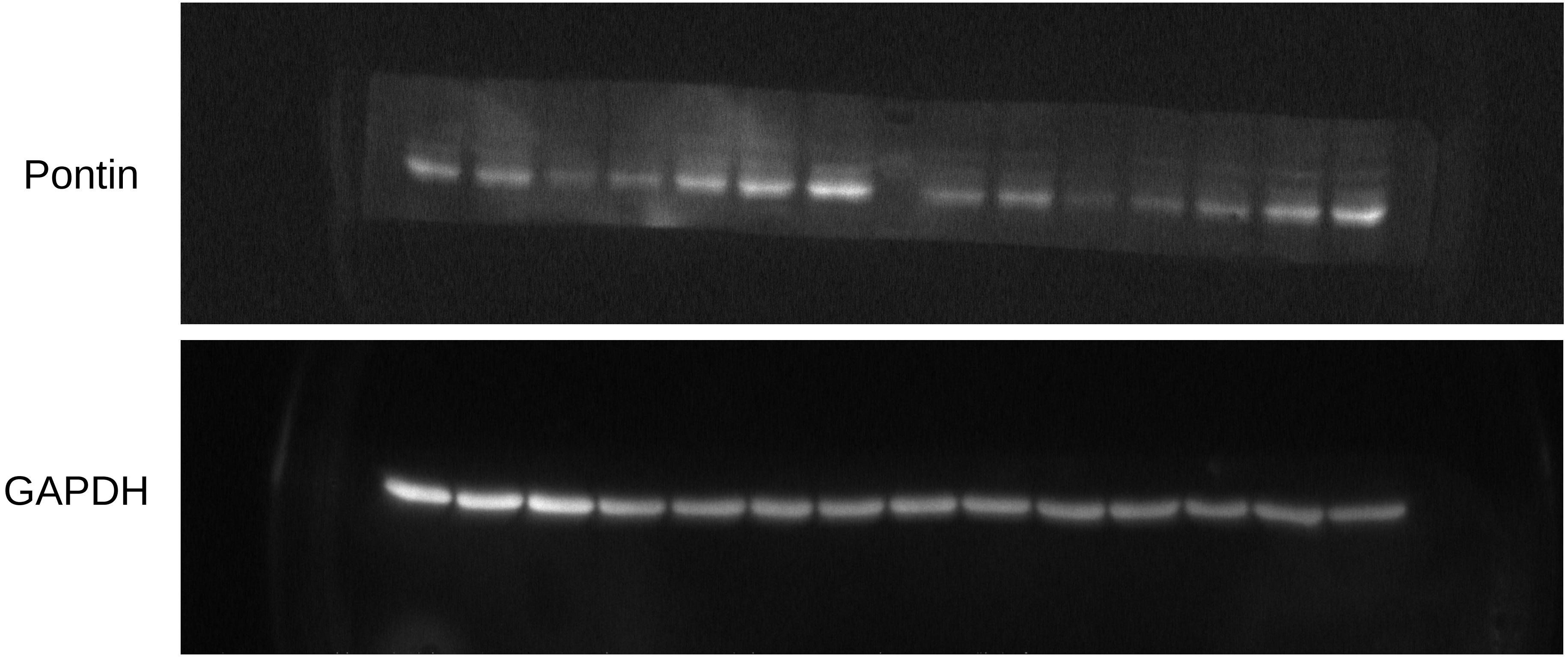
Pontin
GAPDH

Figure 5

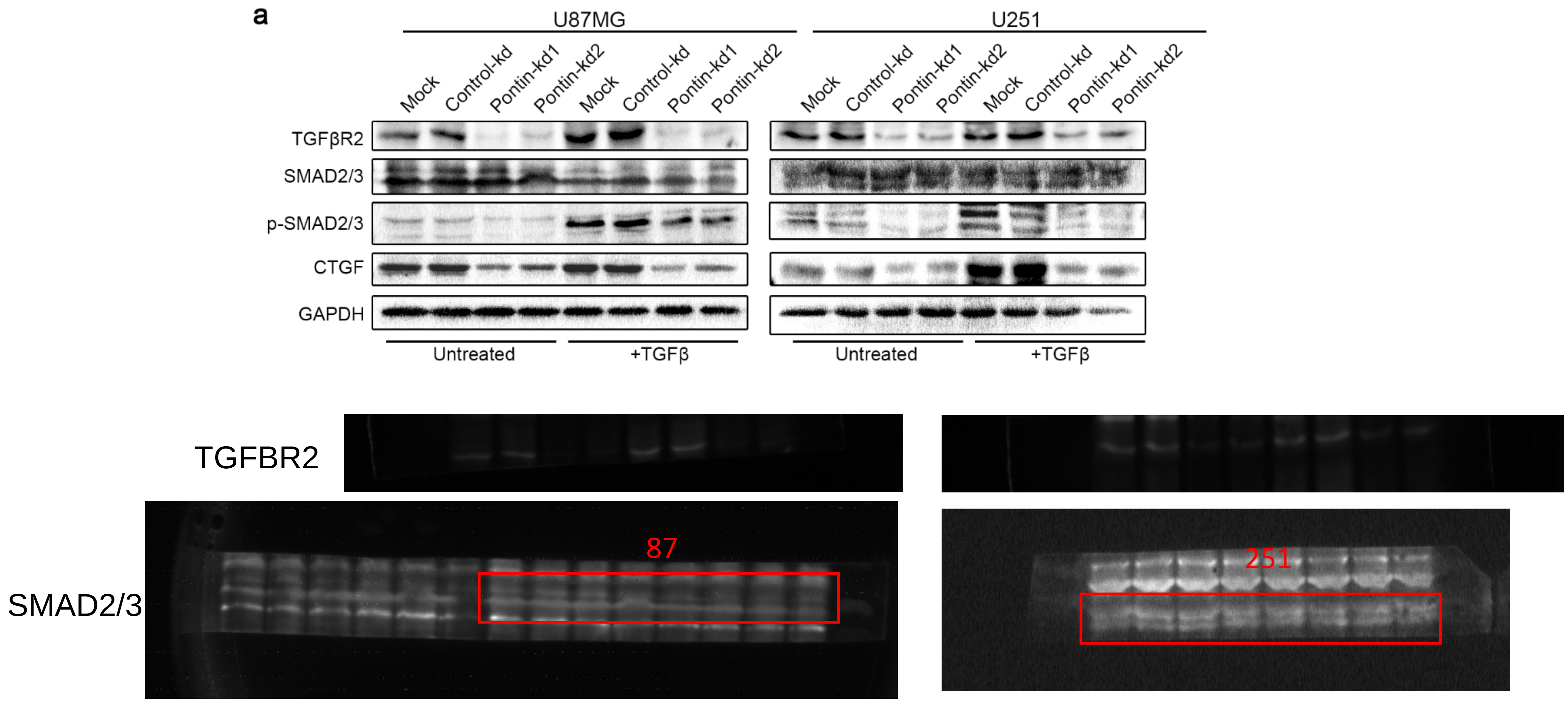
a
U87MG
U251
Mock
Control-kd
Pontin-kd1
Pontin-kd2
TGFβR2
SMAD2/3
p-SMAD2/3
CTGF
GAPDH
Untreated
+TGFβ
TGFBR2
SMAD2/3
87
251

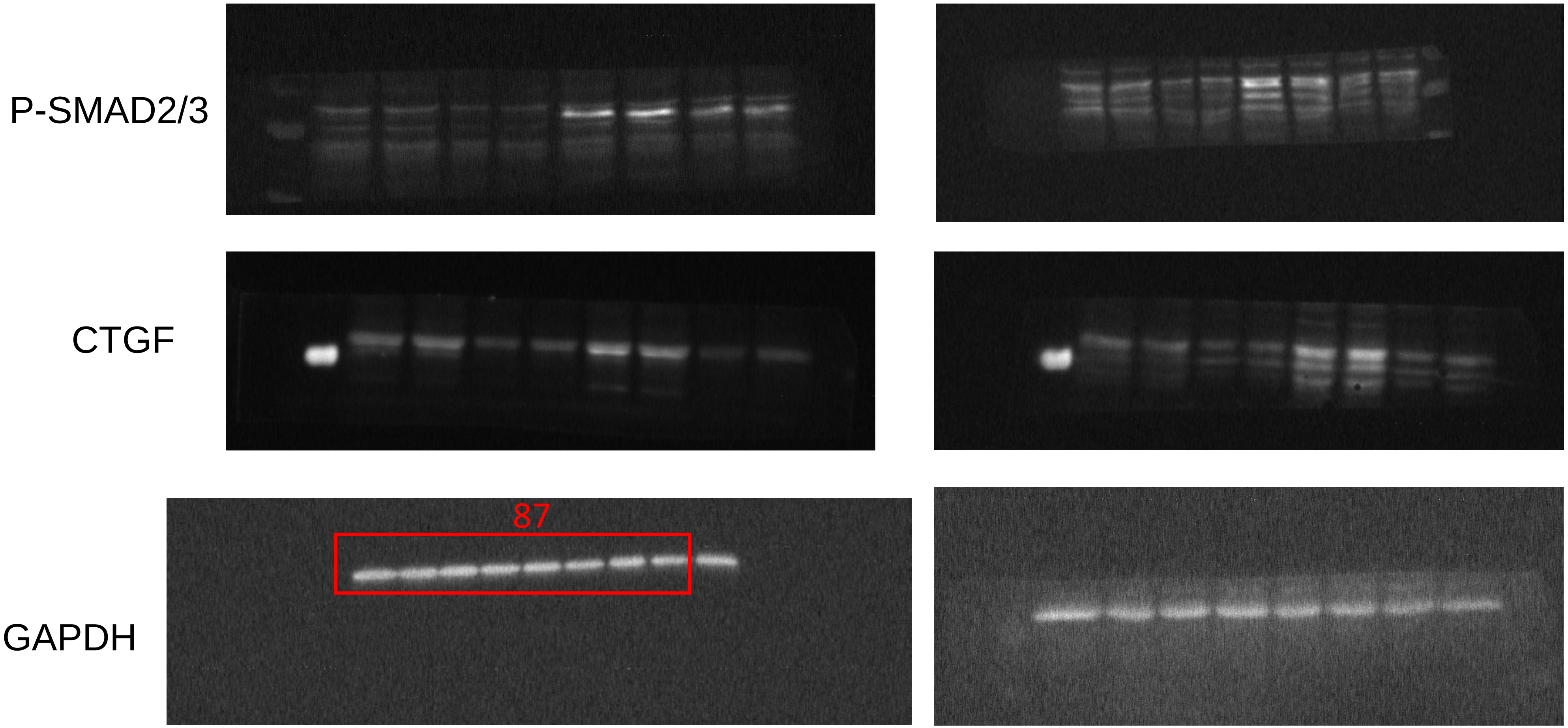
P-SMAD2/3
CTGF
GAPDH
87

Figure 6

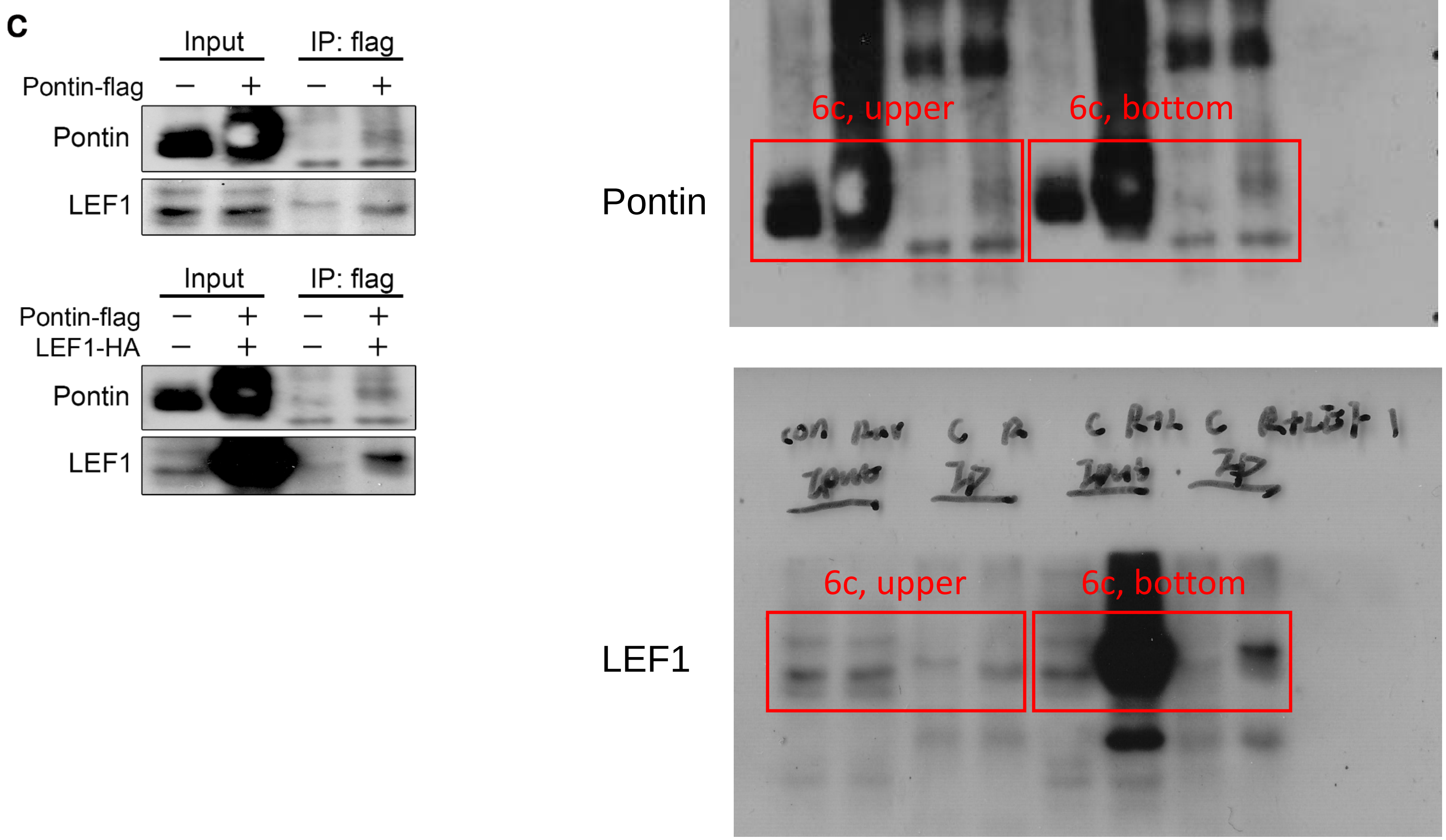
c
Input
IP: flag
Pontin-flag − + − +
Pontin
LEF1
Input
IP: flag
Pontin-flag − + − +
LEF1-HA − + − +
Pontin
LEF1
Pontin
6c, upper
6c, bottom
LEF1
6c, upper
6c, bottom

Figure 7

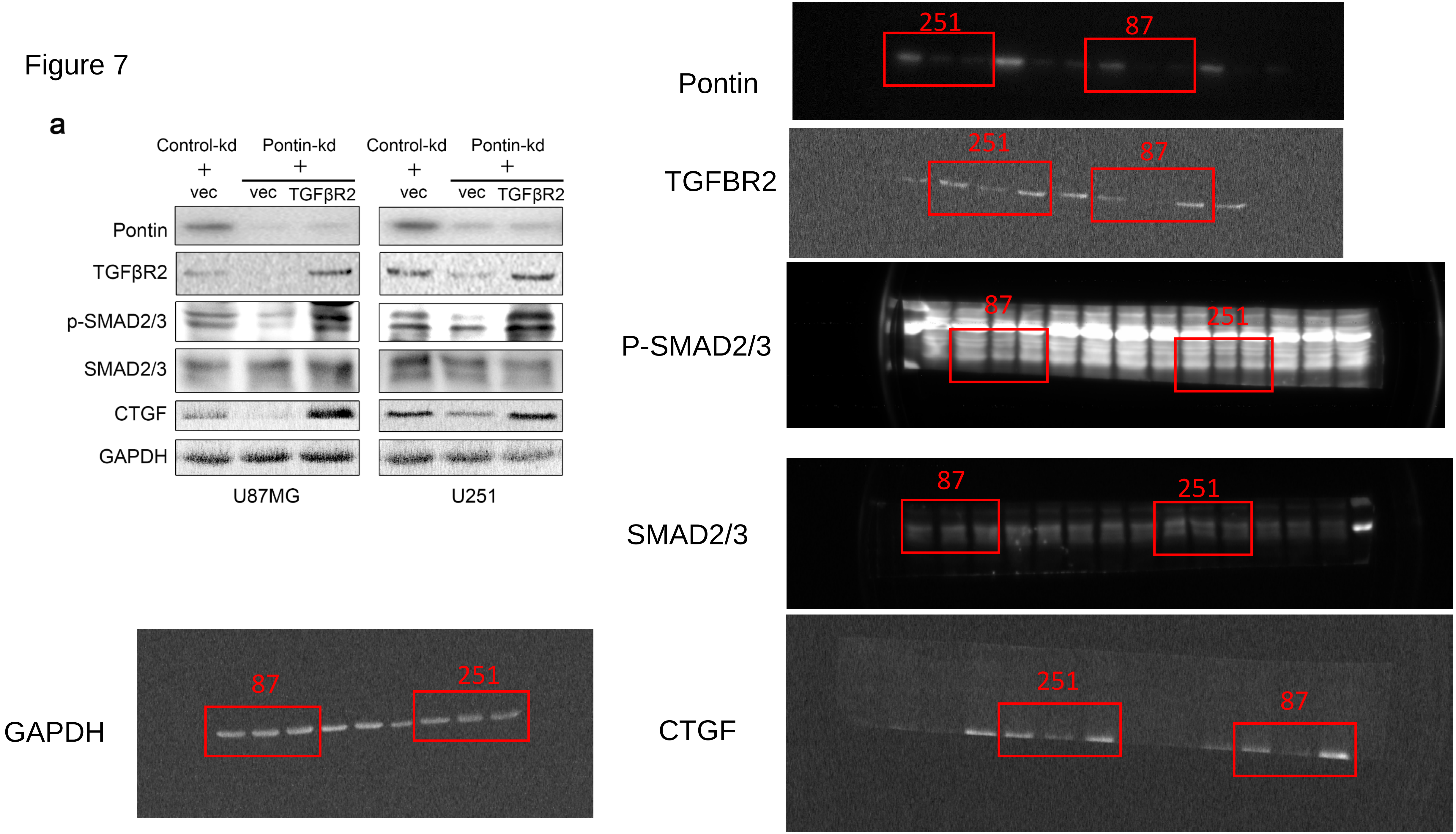
a
Control-kd + vec
Pontin-kd +
vec
TGFβR2
Control-kd + vec
Pontin-kd +
vec
TGFβR2
Pontin
TGFβR2
p-SMAD2/3
SMAD2/3
CTGF
GAPDH
U87MG
U251
Pontin
251
87
TGFBR2
251
87
P-SMAD2/3
87
251
SMAD2/3
87
251
GAPDH
87
251
CTGF
251
87

FigureS1

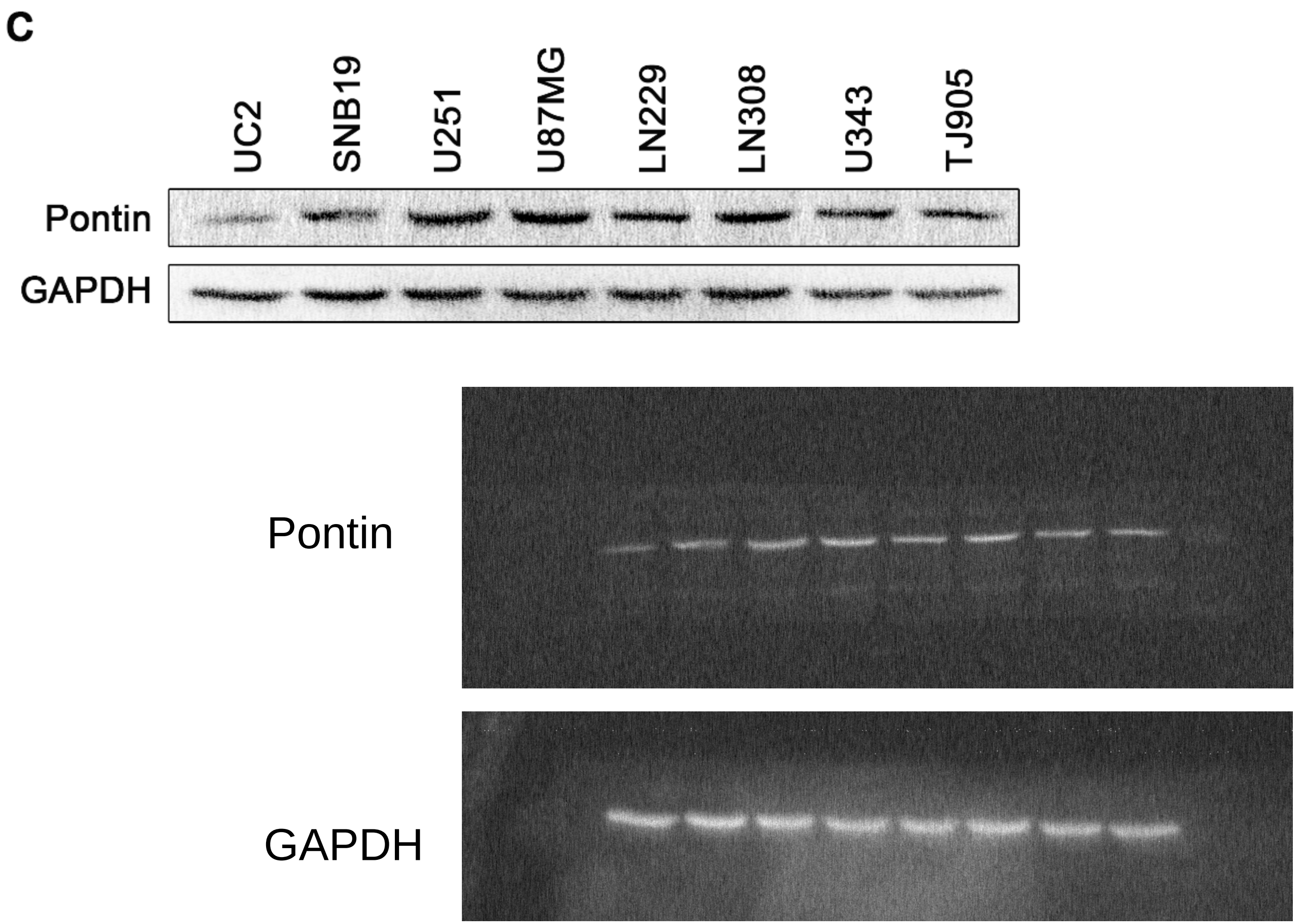
C
UC2
SNB19
U251
U87MG
LN229
LN308
U343
TJ905
Pontin
GAPDH
Pontin
GAPDH

Figure S4

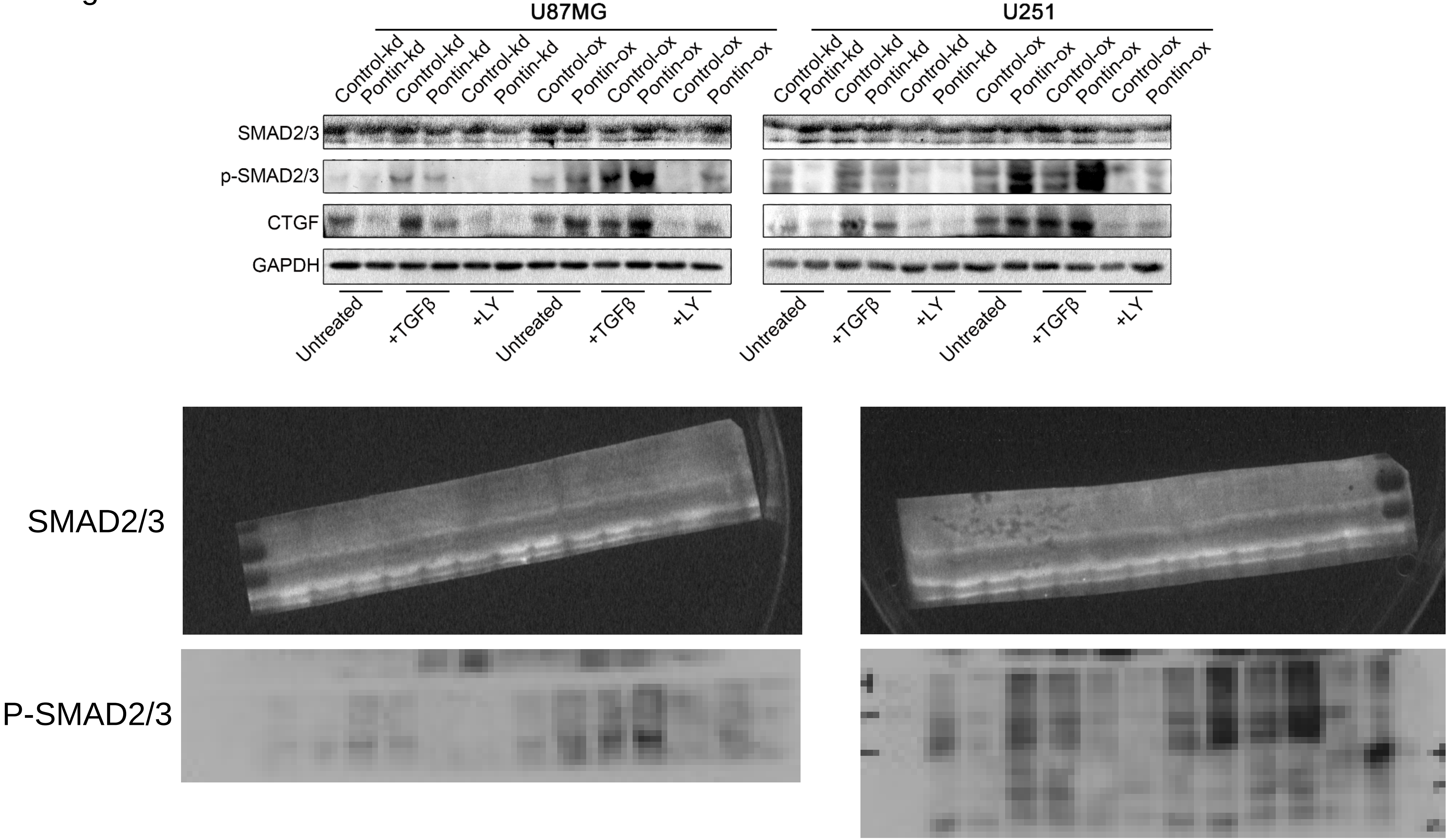

U87MG
U251
Control-kd
Pontin-kd
Control-ox
Pontin-ox
SMAD2/3
p-SMAD2/3
CTGF
GAPDH
Untreated
+TGFβ
+LY
SMAD2/3
P-SMAD2/3

CTGF

GAPDH

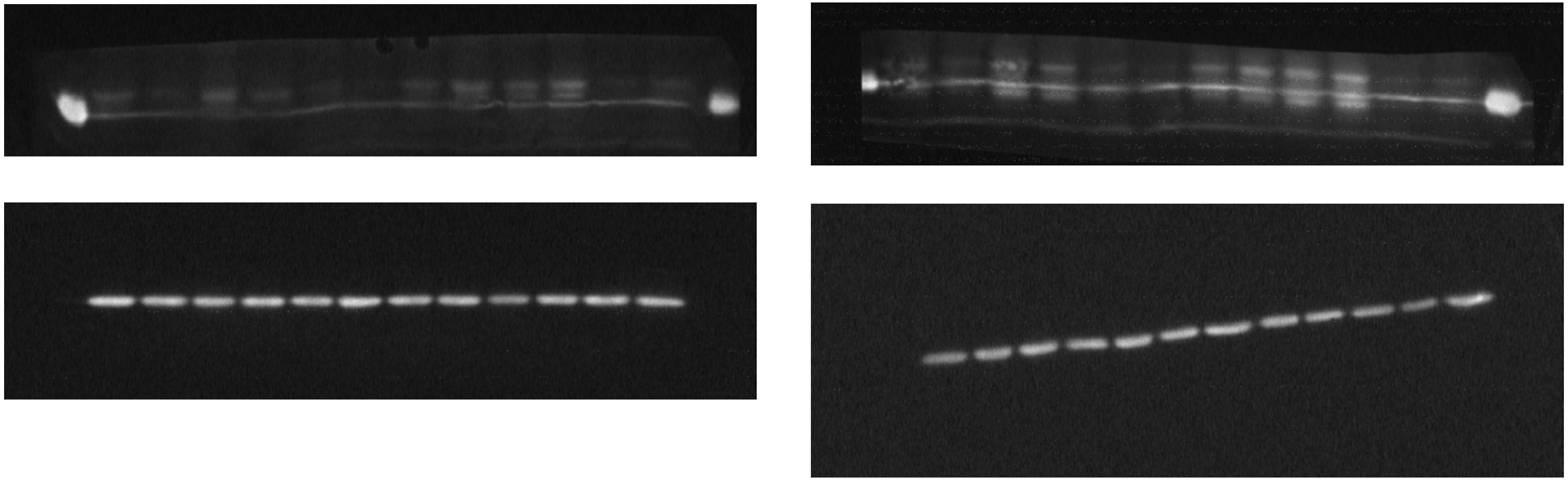

Supplement: Supplementary file 1 — Supplementary materials [file 41419_2022_5265_MOESM1_ESM.pdf]
